# Hybridization Reveals the Evolving Genomic Architecture of Speciation

**DOI:** 10.1016/j.celrep.2013.09.042

**Published:** 2013-10-31

**Authors:** Marcus R. Kronforst, Matthew E.B. Hansen, Nicholas G. Crawford, Jason R. Gallant, Wei Zhang, Rob J. Kulathinal, Durrell D. Kapan, Sean P. Mullen

**Affiliations:** 1 Department of Ecology and Evolution, University of Chicago, Chicago, IL 60637, USA; 2 Department of Biology, Temple University, Philadelphia, PA 19122, USA; 3 Department of Biology, Boston University, Boston, MA 02215, USA; 4 Department of Entomology and Center for Comparative Genomics, California Academy of Sciences, San Francisco, CA 94118, USA; 5 Center for Conservation and Research Training, Pacific Biosciences Research Center, University of Hawaii at Manoa, Honolulu, HI 96822, USA

## Abstract

The rate at which genomes diverge during speciation is unknown, as are the physical dynamics of the process. Here, we compare full genome sequences of 32 butterflies, representing five species from a hybridizing Heliconius butterfly community, to examine genome-wide patterns of introgression and infer how divergence evolves during the speciation process. Our analyses reveal that initial divergence is restricted to a small fraction of the genome, largely clustered around known wing-patterning genes. Over time, divergence evolves rapidly, due primarily to the origin of new divergent regions. Furthermore, divergent genomic regions display signatures of both selection and adaptive introgression, demonstrating the link between microevolutionary processes acting within species and the origin of species across macroevolutionary timescales. Our results provide a uniquely comprehensive portrait of the evolving species boundary due to the role that hybridization plays in reducing the background accumulation of divergence at neutral sites.

## INTRODUCTION

Gene flow prevents the accumulation of genetic differentiation among populations, and as a result, hybridization is often viewed as an impediment to the speciation process (Mayr, 1963). However, increasing evidence across a variety of plant and animal taxa suggests that speciation with gene flow may be more common than previously recognized (Mallet, 2005). Such examples of divergence with gene flow argue for a critical role of divergent selection in the origin of species (Via, 2009). Importantly, these systems also offer an opportunity to identify the genetic changes that underlie species-level divergence, because background differentiation at neutral sites is reduced by persistent hybridization and interspecific gene flow (Nosil et al., 2009; Via, 2009). This approach circumvents a classic problem in the study of speciation: distinguishing the subset of the genome that plays a critical role in the origin of species from the many changes that accumulate after the evolution of reproductive isolation.

Recent studies have documented genome-wide patterns of divergence between closely related sister taxa (Ellegren et al., 2012; Kulathinal et al., 2009; Lawniczak et al., 2010; Nadeau et al., 2013; Neafsey et al., 2010; Staubach et al., 2012; Turner et al., 2005), but the fundamental question of how divergence evolves throughout the process of speciation remains largely unexplored. Theoretical work suggests that divergent genomic regions protect adjacent, tightly linked neutral polymorphism and enhance genetic hitchhiking locally due to reduced migration (Feder et al., 2012a, 2012b; Feder and Nosil, 2010; Nosil et al., 2009). The expected outcome of this is that as phylogenetic distance increases, divergent genomic regions should increase in physical size, leading to reduced genome-wide patterns of gene flow and increased differentiation. This prediction has not been rigorously investigated using whole-genome sequence data, and it remains unclear whether such islands of divergence increase in size, how quickly they grow, or how the number, density, and chromosomal distribution of divergent regions change over time (Feder et al., 2012a; Nadeau et al., 2013; Nosil et al., 2009).

**Figure 1:**
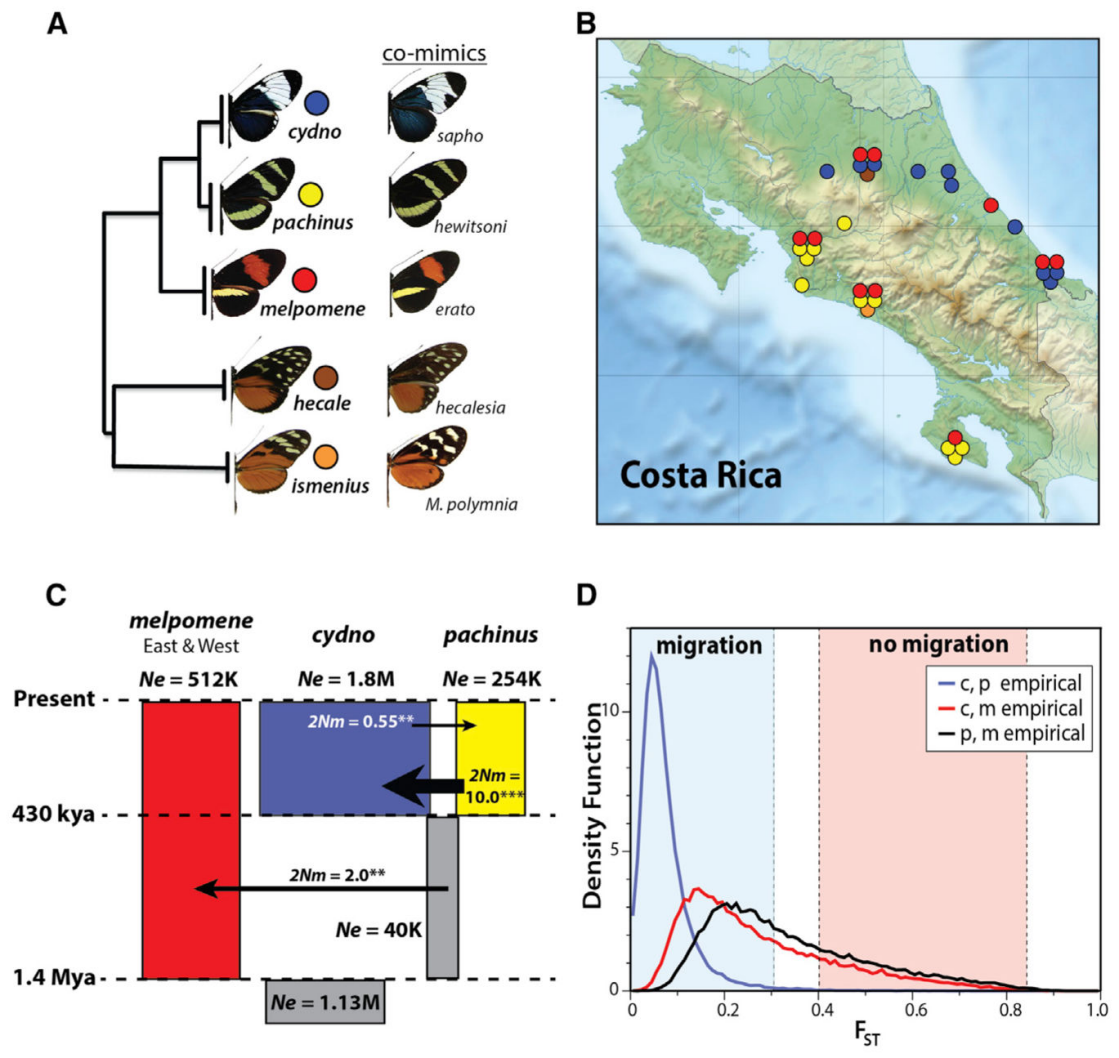
Five Hybridizing Species of *Heliconius* in Costa Rica Demonstrate Varying Levels of Genome-wide Differentiation and Gene Flow (A) Phylogeny of *H. cydno*, *H. pachinus*, and *H. melpomene*, along with their outgroup species, *H. hecale* and *H. ismenius*, based on genome sequence data. Their distantly related comimics are shown on the right. (B) Collection sites of individual samples, color-coded according to (A). (C) History of divergence and gene flow among focal taxa based on analysis of genome-wide data using IMa2 (*N_e_*, effective population size; *2Nm*, population migration rate). (D) Empirical F_ST_ distributions among *H. cydno*, *H. pachinus*, and *H. melpomene*, with shading indicating F_ST_ distributions based on coalescent simulations with and without interspecific gene flow.

The butterfly genus *Heliconius* provides a particularly useful system to explore the dynamics of genome evolution during speciation, because this recent radiation has produced a continuum of co-occurring taxa at different stages of speciation. *Heliconius* is a diverse group of 45 species, well known for bold color patterns and widespread wing-pattern mimicry (Brown, 1981; Joron et al., 2006a; Papa et al., 2008; Sheppard et al., 1985). Across the Neotropics, local *Heliconius* communities generally consist of 10 to 15 species, with four or five of these coming from a subclade of closely related species that are known to hybridize (Mallet et al., 2007). In Costa Rica, the hybridizing *Heliconius* community consists of five species (Figure 1A); sister species *H. cydno* and *H. pachinus* are restricted to opposite coastal drainages with a contact zone in the center of the country, while *H. melpomene*, *H. hecale*, and *H. ismenius* are distributed throughout (Figure 1B).

These species represent different points on the trajectory of speciation (Mallet et al., 1998; Merrill et al., 2011). For instance, *H. cydno* and *H. pachinus* are closely related, ecologically similar species that are completely interfertile, producing viable, fertile hybrids in captivity (Gilbert, 2003; Kronforst et al., 2006a, 2006c). In nature, however, there is pronounced reproductive isolation between them, mediated by a combination of their largely parapatric distributions, divergent mimicry phenotypes that generate extrinsic postzygotic isolation, and strong assortative mate preferences that generate sexual isolation (Kronforst and Gilbert, 2008; Kronforst et al., 2007a, 2007b, 2006c). *Heliconius melpomene* is sympatric with *H. cydno* on Costa Rica’s Caribbean drainage and it is sympatric with *H. pachinus* on the Pacific drainage. Comparison of *H. melpomene* to either *H. cydno* or *H. pachinus* represents a further step in the process of speciation (Mallet et al., 1998, 2011). In addition to divergent mimicry phenotypes (Merrill et al., 2012) and strong sexual isolation (Jiggins et al., 2001), *H. melpomene* and *H. cydno*/*pachinus* are also ecologically and behaviorally distinct (Benson, 1978; Estrada and Jiggins, 2002; Mallet and Gilbert, 1995; Smiley, 1978), and crosses between them result in Z-linked female sterility (Naisbit et al., 2002) and disruptive sexual selection against hybrids (Naisbit et al., 2001). Yet, despite strong reproductive isolation among species, they are all known to hybridize (Mallet et al., 2007), and previous analyses suggest ongoing gene flow throughout the process of speciation (Beltrán et al., 2002; Bull et al., 2006; Kronforst et al., 2006b, 2008; Martin et al., 2013).

Recent genetic work in this subclade of *Heliconius* has focused on characterizing the molecular basis of wing-pattern mimicry (Baxter et al., 2010; Joron et al., 2006b; Martin et al., 2012; Reed et al., 2011) and then examining signatures of genetic differentiation and introgression around these mimicry genes (Baxter et al., 2010; Chamberlain et al., 2011; Heliconius Genome Consortium, 2012; Nadeau et al., 2012; Pardo-Diaz et al., 2012; Reed et al., 2011). The results of this work indicate that DNA sequence variation around mimicry genes is strongly differentiated between species and subspecies with divergent mimicry phenotypes, and there is evidence that mimicry alleles have introgressed between phenotypically similar species. However, population genomic analyses outside of these mimicry genes have had less resolution because they have utilized small samples sizes and looked at only a small fraction of the genome, using either targeted sequencing of a few regions of the genome (Nadeau et al., 2012), widely spaced molecular markers (Nadeau et al., 2013), or a combination of the two (Heliconius Genome Consortium, 2012).

The recent publication of a reference genome sequence for *H. melpomene* (Heliconius Genome Consortium, 2012) now enables full genome characterization of genetic variation in *Heliconius*, permitting a complete census of genome-wide divergence associated with speciation. Here, we present whole-genome resequencing data for five sympatric hybridizing taxa with divergent mimetic wing patterns to examine how genome divergence is initiated and how it evolves over time during the process of speciation with gene flow. Our results indicate that (1) divergent natural selection acts first on a handful of color-patterning loci, triggering population divergence leading to speciation in *Heliconius*; (2) the species boundary subsequently evolves very rapidly across the entire genome primarily due to the origin of newly divergent regions; and (3) patterns of molecular variation across the genome reflect a dynamic interplay between selection and gene flow.

## RESULTS AND DISCUSSION

### Substantial Interspecific Gene Flow Reduces Background Divergence among Species

Hybridization and gene flow among *Heliconius* species is well documented. Sympatric species from across our focal clade hybridize at appreciable frequencies in nature and hybrids that have been collected include both F1 and backcross hybrids (Mallet et al., 2007; Mavárez et al., 2006). Furthermore, advanced generation hybrids are common. Our previous work on the hybridizing community in Costa Rica revealed that a number of field-collected *H. cydno*, *H. pachinus*, and *H. melpomene* individuals had mixed ancestry (Kronforst, 2008; Kronforst et al., 2006b), indicating a relatively recent hybrid ancestor (Figure S1A). This hybridization appears to have resulted in long-term introgression among species as previous studies have routinely documented strong statistical evidence for interspecific gene flow (Bull et al., 2006; Kronforst, 2008; Kronforst et al., 2006b; Martin et al., 2013). In addition, there is good genetic support for (1) hybrid ancestry of field-collected individuals with recombinant wing patterns (Dasmahapatra et al., 2007), (2) at least one instance of hybrid speciation (Jiggins et al., 2008; Mavárez et al., 2006; Salazar et al., 2010), and (3) multiple instances of introgression of wing-patterning alleles across the species boundary (Heliconius Genome Consortium, 2012; Pardo-Diaz et al., 2012; Smith and Kronforst, 2013).

To examine genome-wide patterns of introgression and divergence, we sequenced the genomes of ten wild-caught samples from each of our three focal species, *H. cydno*, *H. pachinus*, and *H. melpomene*, as well as one sample from each of the two closely related outgroup species, *H. hecale* and *H. ismenius*. Each sample was sequenced to an average depth of 16× using an Illumina Hi-Seq 2000 (Tables S1 and S2). We mapped the data for each sample back to the *H. melpomene* reference genome (Heliconius Genome Consortium, 2012) and scored polymorphisms using the GATK (DePristo et al., 2011). Our final data set consisted of approximately 33 million SNPs, covering the entire genome, with over 97% of these covered in each sample (Table S2). Importantly, we selected samples for sequencing that did not show evidence of recent mixed ancestry (Figure S1A) so as to not bias our estimates of interspecific gene flow. We subsequently verified that our sequenced samples showed no recent admixture using our genome-wide SNP data (Figure S1B).

As a first step in characterizing this system, we used the isolation-with-migration model (IMa2), incorporating data from many loci sampled across the genome, to estimate the history of divergence and gene flow among species (Figure 1C; Table S3). The inferred divergence times and migration rates among species are consistent with previous results based on smaller data sets (Bull et al., 2006; Kronforst, 2008; Kronforst et al., 2006b). We further characterized the inferred demographic parameter estimates by simulating genome-scale data, with and without inter-specific gene flow. Simulations including persistent interspecific gene flow yielded divergence levels similar to our observed data, whereas simulations without gene flow yielded divergence levels five to six times greater than observed (Figure 1D). Together, these results suggest that rates of gene flow among species are high and sufficient to prevent the strong, neutral genetic differentiation we would expect in the absence of introgression. In other words, interspecific gene flow appears to be partially homogenizing genetic variation in portions of the genome that are free to cross the species boundary, permitting a comprehensive investigation of how species-level divergence is initiated at the genomic level and how it subsequently evolves.

**Figure 2:**
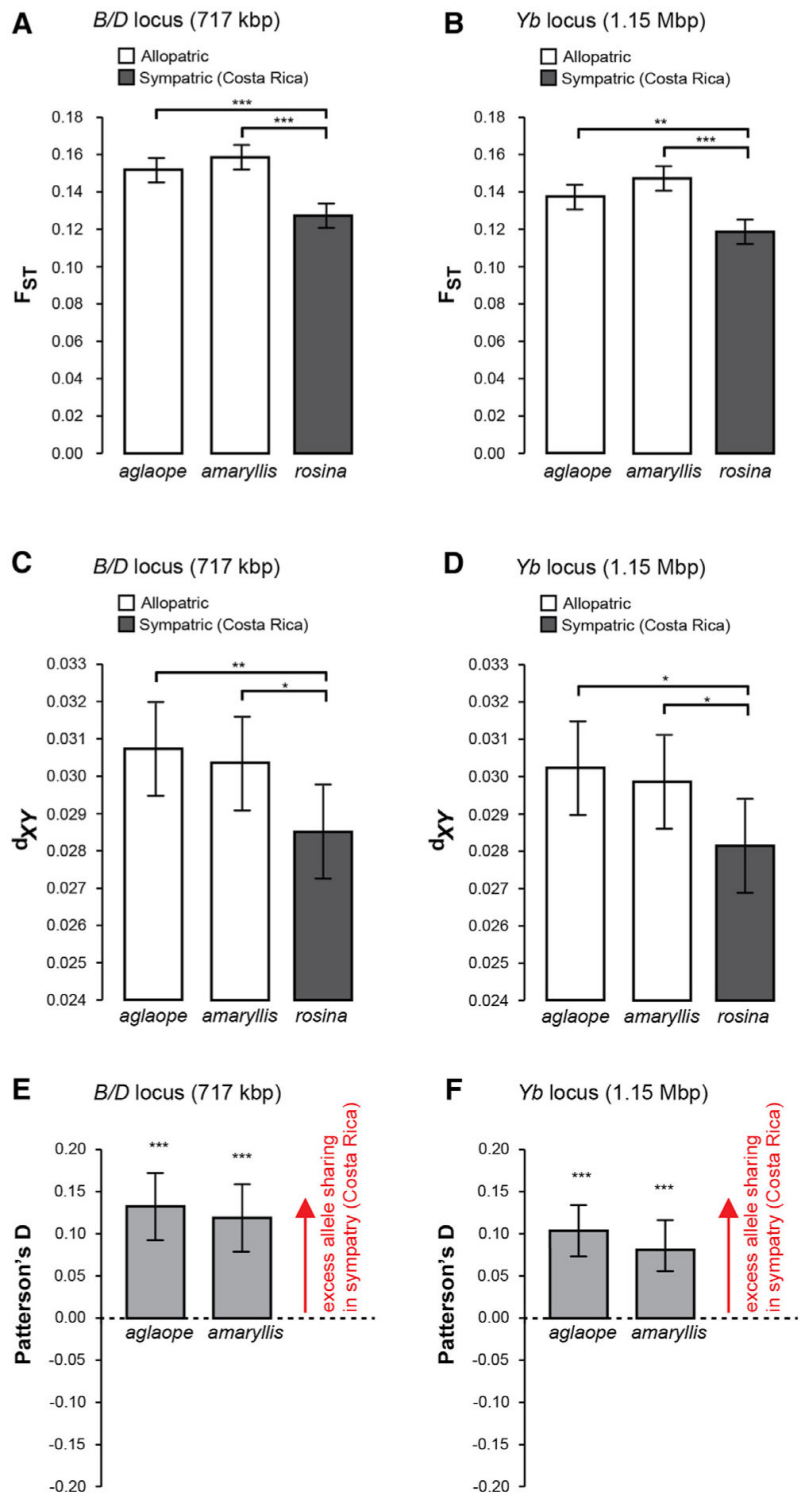
Additional Evidence for Gene Flow among Sympatric Species in Costa Rica (A–D) Sympatric *H. melpomene* and *H. cydno* show reduced divergence, measured by both F_ST_ and *d_XY_*, relative to allopatric comparisons, across two different regions of the genome. Error bars (indicating 95% confidence intervals) and p values are based on bootstrap resampling. (E and F) Furthermore, Patterson’s D statistic is highly elevated in these regions, indicative of biased allele sharing in sympatry due to introgression. Error bars (indicating 95% confidence intervals) and p values are based on bootstrap resampling. *p < 0.05, **p < 0.01, ***p < 0.001.

To test this hypothesis, and further document the influence of interspecific gene flow among sympatric species in Costa Rica, we compared measures of genetic divergence and allele sharing between *H. cydno* from Costa Rica and three different populations of *H. melpomene*: sympatric *H. melpomene rosina* from Costa Rica, allopatric *H. melpomene aglaope* from Peru, and allopatric *H. melpomene amaryllis* from Peru (Figure 2). The allopatric *H. melpomene* data consist of approximately 1.8 Mbp of sequence data around two mimicry loci, *B/D* and *Yb*, from four samples of each Peruvian population, which were sequenced as part of the *Heliconius* Genome Project (Heliconius Genome Consortium, 2012). The results reveal that for two different estimates of genetic divergence, F_ST_ and *d_XY_*, sympatric *H. melpomene* and *H. cydno* were more similar (Figures 2A–2D). Furthermore, by using Patterson’s D statistic (Durand et al., 2011) to compare patterns of derived allele sharing between populations, we found a substantial enrichment of shared derived alleles in sympatric comparisons relative to allopatric comparisons (Figures 2E and 2F), indicative of local introgression. Unlike the adaptive introgression of mimicry documented between other taxa at the *B/D* and *Yb* loci (Heliconius Genome Consortium, 2012; Pardo-Diaz et al., 2012; Smith and Kronforst, 2013), the signatures of gene flow we detected here between *H. melpomene* and *H. cydno* are not related to mimicry introgression because the two species show highly divergent phenotypes at both mimicry loci. It is important to note that these results only hint at the real rates of interspecific gene flow for three reasons. First, this analysis is based on examining sequence variation around mimicry loci, which are under divergent selection between *H. melpomene* and *H. cydno* in Costa Rica and should be (and are) resistant to interspecific gene flow (see below). Hence, the evidence for gene flow we found in these regions is likely to be much more modest than regions of the genome not linked to divergent mimicry loci. Second, we can only document gene flow that has occurred since the subspecies of *H. melpomene* split from one another, which is recent relative to the split between *H. melpomene* and *H. cydno*. Therefore, a longer history of introgression is lost in these analyses. Third, *H. melpomene aglaope* and *amaryllis* have both experienced substantial gene flow with a close relative of *H. cydno*, *H. timareta*, at the *B/D* and *Yb* loci (Heliconius Genome Consortium, 2012; Pardo-Diaz et al., 2012; Smith and Kronforst, 2013). Therefore, our allopatric *melpomene* have potentially experienced the same homogenizing effect with a *cydno*-like genome, which will artificially decrease allopatric F_ST_ and *d_XY_* estimates as well as Patterson’s D.

### Genome Divergence at the Earliest Stage of Speciation Centers on Mimicry Genes

**Figure 3:**
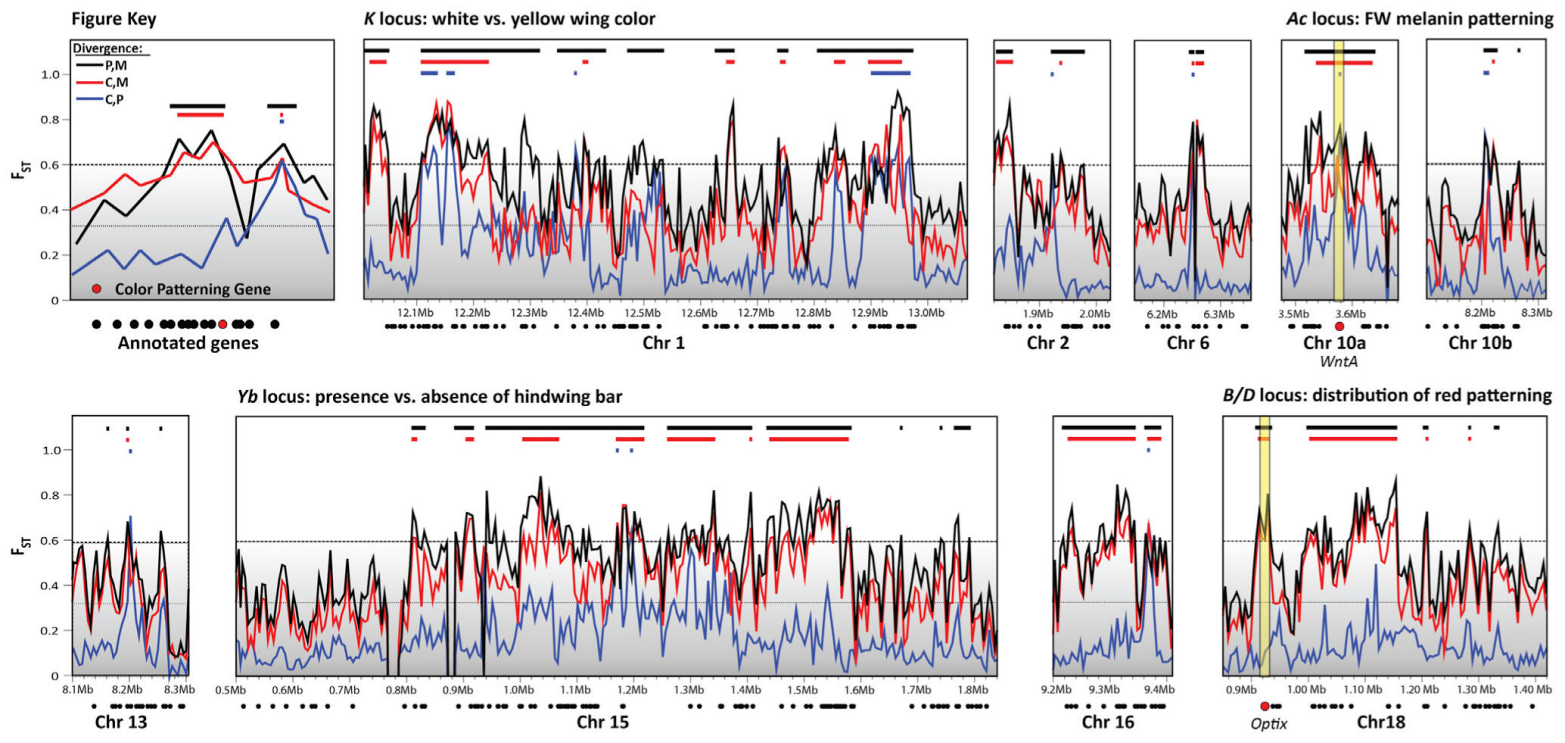
Signatures of Genomic Differentiation, Focusing on the 12 Regions that Are Divergent between *H. cydno* and *H. pachinus* Known wing color patterning loci (*K*, *Ac*, *Yb*, *B/D*) are listed, as are genes *WntA* and *Optix*. F_ST_ plots and divergent segment markers are color coded by pairwise comparison.

We examined the genome-wide distribution of genetic divergence in pairwise comparisons among sympatric *H. cydno*, *H. pachinus*, and *H. melpomene* from Costa Rica. For these analyses, we calculated genetic differentiation, analysis of molecular variance (AMOVA)-based F_ST_ (Excoffier et al., 1992), for 5 kbp windows covering the entire genome and identified outliers using an empirically derived significance threshold (Figure S2). Because adjacent windows showing significant differentiation are not biologically independent (see Experimental Procedures), they were connected into larger divergent segments. Surprisingly, the comparison between the most closely related species, *H. cydno* and *H. pachinus*, revealed only 12 narrow (mean = 14 kbp) divergent regions across the genome, spanning a total of 165 kbp (Figure 3). These regions were so narrow, in fact, that they could have been missed in previous restriction-site-associated DNA (RAD) studies (Heliconius Genome Consortium, 2012; Nadeau et al., 2013), because the average marker spacing of *Heliconius* RADs has been between 27 and 39 kbp (Nadeau et al., 2013).

The distribution of divergent regions between *H. cydno* and *H. pachinus* was highly nonrandom (Fisher’s exact test, p < 0.01; Figure S3), with eight of them mapping to the locations of known mimicry genes (Baxter et al., 2010; Chamberlain et al., 2011; Kronforst et al., 2006a, 2006c; Martin et al., 2012; Reed et al., 2011). For instance, 4 of the 12 divergent regions sit within 1 Mbp of one another on chromosome 1, in the location of a locus that controls wing color and mate preference in *H. cydno* and *H. pachinus* (Chamberlain et al., 2009; Kronforst et al., 2006c). Similarly, two divergent regions are located on chromosome 10, near the gene *WntA*, which controls melanin patterning on the forewing (Martin et al., 2012). Two additional divergent regions are on chromosome 15, in the location of the mimicry locus that controls melanin patterning on the hindwing (Joron et al., 2006b). There is a signal of enhanced differentiation around the gene *optix*, which controls red patterning in *Heliconius* (Reed et al., 2011), but it did not pass the significance threshold in the comparison between *H. cydno* and *H. pachinus*, both of which lack striking red coloration. However, it is important to note that there was significant divergence in and around *optix* in both comparisons with red-winged *H. melpomene*, which are the comparisons that have radically different alleles at this mimicry locus.

These results suggest a central role for mimicry evolution in promoting the earliest stages of speciation in *Heliconius*. This finding matches well with previous research on *Heliconius* showing that mimetic wing patterns experience strong divergent natural selection (Kapan, 2001; Mallet et al., 1990; Mallet and Barton, 1989) and that shifts in wing pattern generate reproductive isolation, both premating and extrinsic postzygotic (Chamberlain et al., 2009; Jiggins et al., 2001; Kronforst et al., 2006c; Merrill et al., 2011, 2012; Naisbit et al., 2001). The extent to which our genome-scan results overlap with previous ecological and behavioral research as well as recent positional cloning of mimicry loci is remarkable, and the intersection of these various forms of data provide compelling evidence for ecological speciation in *Heliconius* butterflies. While previous work has documented divergence around mimicry genes in *Heliconius* (Nadeau et al., 2012), our unbiased survey of the entire genome allows us to show that these loci do genuinely stand out from the rest of the genome as the initial targets of selection that then precipitate speciation.

**Figure 4:**
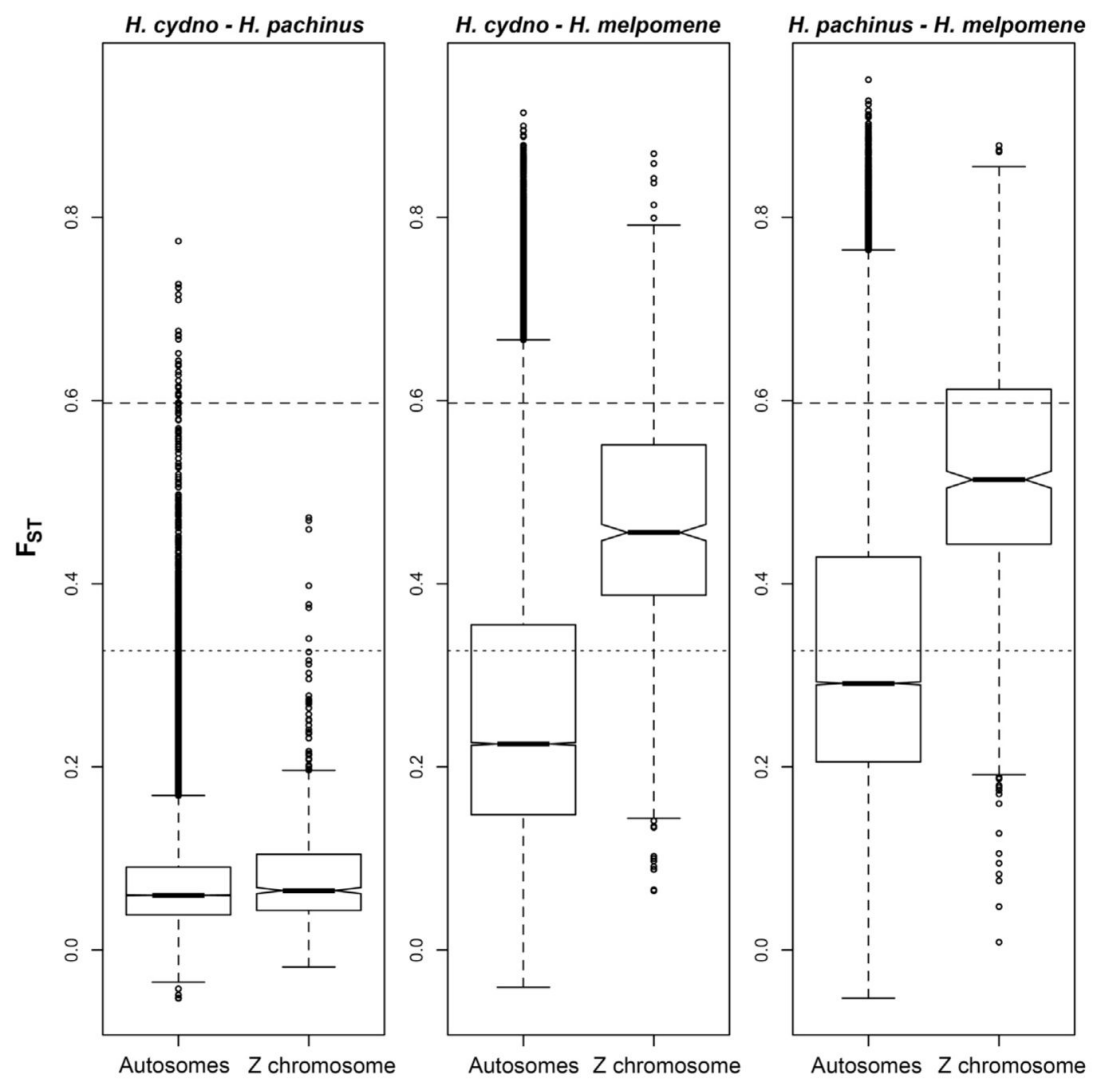
Z Chromosome and Autosome Divergence in Pairwise Comparisons between Species Pairwise F_ST_ represented as boxplots with whiskers between (1) *cydno-pachnius* (left), (2) *cydno-melpomene* (middle), and (3) *pachinus-melpomene* (right) for autosomes versus the Z chromosome, highlighting elevated divergence on the Z chromosome in comparisons with *H. melpomene*. Similar distributions, separated out by chromosome, are shown in Figure S7.

The few highly divergent regions not linked to mimicry loci suggest additional genes that are likely to play an important role in the early stages of speciation. These four regions contain only six genes: the fatty acid synthase gene *p260* on chromosome 2, *abl-interactor 2* on chromosome 6, a fatty acid elongase gene on chromosome 13, and three clustered genes on chromosome 16 (a cytoplasmic dynein 1 intermediate chain gene similar to *short wing* in *Drosophila*, a peptide deformylase gene, and *3-hydroxyisobutyryl-coenzyme A hydrolase*). Interestingly, chromosomal inversions and the Z (sex) chromosome do not appear to play a role in maintaining this young species boundary (Table S4; Figure 4), suggesting that these factors emerge later in *Heliconius* speciation, following initial ecological divergence.

### Genome-wide Divergence Grows Rapidly, Primarily due to the Origin of Newly Divergent Regions

We next examined how genome-wide divergence evolves over time. Pairwise comparisons between *H. melpomene* and either *H. cydno* or *H. pachinus* revealed 100 to 200 times more divergence, with the cumulative portion of the genome showing significant differentiation increasing from 165 kbp in the *cydno*/*pachinus* comparison to 19 Mbp and 33 Mbp in the two comparisons with *H. melpomene* (Table 1). The two comparisons with *H. melpomene* are not phylogenetically independent, but the comparison between *H. cydno* and *H. pachinus* is independent of the comparison between their common ancestor and *H. melpomene*. Given that only approximately 1 million years separates these divergence events, the sizeable divergence in comparisons with *H. melpomene* appears to be much more than that predicted by the modest divergence between *H. cydno* and *H. pachinus*. This result suggests a nonlinear relationship between time since speciation and the accumulation of genome-wide divergence.

**Figure 5:**
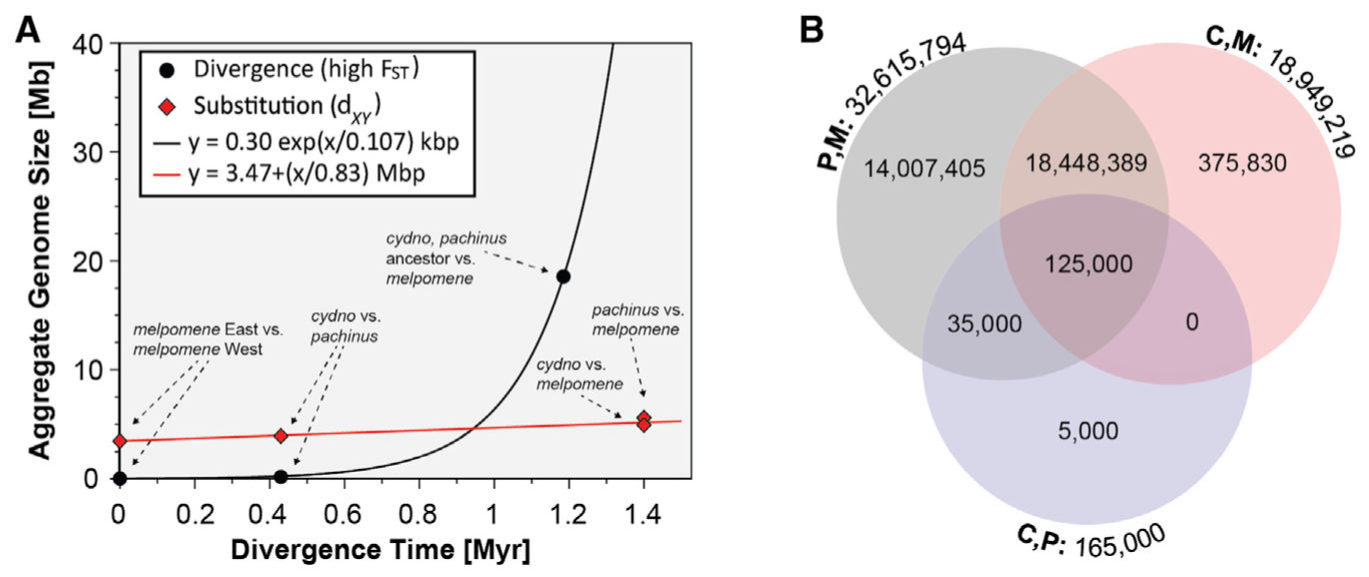
Dynamics of Genome-wide Divergence during Speciation (A) Exponential growth in genome-wide divergence compared to linear substitutions as a function of divergence time. Note that *d_XY_* is expressed as the total number of nucleotide substitutions across the genome, rather than a proportion, so the same y axis applies to both the divergence and substitution lines. (B) Venn diagram of the total base-pair overlap between divergent regions in pairwise comparisons.

To examine the evolution of divergence further, we separated our *H. melpomene* samples into two populations: one from the Caribbean drainage (east) and one from the Pacific drainage (west), and we compared them to estimate the amount of genome divergence for a within-species comparison. This intraspecific comparison yielded a single, 10 kbp divergent region that distinguished Caribbean *H. melpomene* from Pacific *H. melpomene*. We also estimated DNA sequence divergence in all comparisons as mean *d_XY_*. We then plotted the aggregate portion of the genome contained in highly divergent regions, as a function of time since divergence, for the following comparisons: *melpomene* east versus *melpomene* west, *cydno* versus *pachinus*, *and melpomene* versus the common ancestor of *cydno* and pachinus (estimated as the subset of highly divergent regions shared between *melpomene* versus *cydno* and *melpomene* versus *pachinus* comparisons). This yielded three phylogenetically independent comparisons. We also plotted mean *d_XY_* for the following comparisons: *melpomene* east versus *melpomene* west, *cydno* versus *pachinus*, *melpomene* versus *cydno*, and *melpomene* versus *pachinus*. Given the divergence time estimates, this analysis indicates that genome-wide divergence accumulates slowly then rapidly rises, despite a constant substitution rate (Figure 5A). The observed relationship hinges on how genome-wide differentiation occurs during the earliest stages of speciation when phenotypic and behavioral differences are apparent but most of the genome has not yet diverged. Our data suggest that an exponential model is more likely than a linear one (Akaike information criterion [AIC] = 9.06 versus 61.7, 2 df). We explored this same phenomenon using a separate approach, counting the number of fixed differences in pairwise comparisons. Here too, we see evidence for a nonlinear accumulation of genetic differentiation (Table S5). Our results are also consistent with a step change, whereby divergence shifts rapidly from low to high levels, but more data points will be required to determine the exact shape of this function.

Why do the rates of accumulation for fixed differences and highly differentiated portions of the genome increase over evolutionary time? We suspect that this is a direct consequence of the interspecific gene flow we have documented and how this parameter changes over time. Specifically, our results suggest that rates of hybridization and introgression decrease with time during the speciation process, as expected. The patterns we observe suggest that there is a tipping point in the rate of inter-specific gene flow, below which its homogenizing effect is overwhelmed by other evolutionary processes. Hence, much of the genome remains quite similar for an extended period of time following initial divergence due to gene flow, but then genome-wide differentiation grows explosively later in the speciation process. Interestingly, the apparent exponential growth of genome-wide divergence found here reflects what has been shown for at least one byproduct of genome divergence: the accumulation of intrinsic postzygotic incompatibilities (Matute et al., 2010; Moyle and Nakazato, 2010).

Traditionally, the snowball effect for hybrid incompatibilities has been interpreted as a product of the nonlinear accumulation of epistatic interactions that are expected to result from a linear gene substitution process. While tentative, our results raise the intriguing possibility that a second phenomenon, the nonlinear rate of genome divergence, may also contribute to this snowball effect. It remains to be seen whether our observation of exponential growth holds up as additional data points are added, whether this is a general phenomenon or one that only applies to systems experiencing divergence with gene flow, and what is ultimately responsible for the phenomenon.

Our results revealed a high degree of overlap in the divergent regions across all comparisons (Figure 5B). While these comparisons are not independent, the fact that almost all of the divergent regions between closely related *H. cydno* and *H. pachinus* are also divergent in comparisons with *H. melpomene* suggests that the process of divergence is repeatable. Furthermore, while islands of divergence do grow over time, they remain quite narrow, such that the vast majority of increased genomic divergence in comparisons with *H. melpomene* results from the origin of new divergent regions (Table 1). This result is in contrast to a divergence hitchhiking model of speciation with gene flow whereby genome-wide divergence is achieved by expansion in the physical size of initial islands of divergence. The rapid origin of new divergent regions appears to be partially driven by selection (see below), but it also may be influenced by genomic hitchhiking, whereby genome-wide divergence is facilitated by reductions in gene flow resulting from divergent selection. This conclusion remains to be tested further but, intriguingly, while we found that divergent regions were distributed nonrandomly in the genome when comparing *H. cydno* and *H. pachinus*, comparisons with *H. melpomene* revealed no clustering of divergent regions among chromosomes (p > 0.61 in both comparisons), except on the Z chromosome, which exhibited enhanced divergence in comparisons with *H. melpomene* (Figure 4). Enhanced divergence on the Z chromosome is consistent with both a neutral process, whereby this chromosome diverges faster as a result of its reduced effective population size and the fact that an important component of reproductive isolation, hybrid female sterility, is Z linked in crosses between *H. melpomene* and *H. cydno* (Naisbit et al., 2002). Finally, we found that gene content across all divergent regions was enriched for a variety of Gene Ontology (GO) terms, including categories that are likely to be important in the evolutionary history of *Heliconius*, such as vision, learning, and morphogenesis (Table S6).

### Genome Divergence Associated with Speciation Is Fueled by Selection and Adaptive Introgression

Given the history of interspecific gene flow among species, what is responsible for observed divergence between species? One possibility is that F_ST_ outliers are driven primarily by linked selection, including processes such as genetic hitchhiking and background selection, which will reduce intraspecific diversity and elevate F_ST_. However, this predicts that regions of high F_ST_ should localize to regions of the genome with reduced recombination. In contrast to this prediction, our previous genetic mapping results (Kronforst et al., 2006a, 2006c) reveal that mimicry loci, which are the first regions to diverge during speciation, are not in regions of low recombination (Figure S4). Rather, we hypothesize that observed genome divergence exists because of natural (Kapan, 2001; Mallet et al., 1990; Mallet and Barton, 1989; Merrill et al., 2012) and sexual selection (Chamberlain et al., 2009; Jiggins et al., 2001; Kronforst et al., 2006c; Naisbit et al., 2001). Furthermore, the evolution of mimicry proceeds by initial, strong divergent selection followed by long-term purifying selection. If divergent genome regions generally behave like the mimicry loci, we might expect to see the combined actions of both divergent and purifying selection.

**Figure 6:**
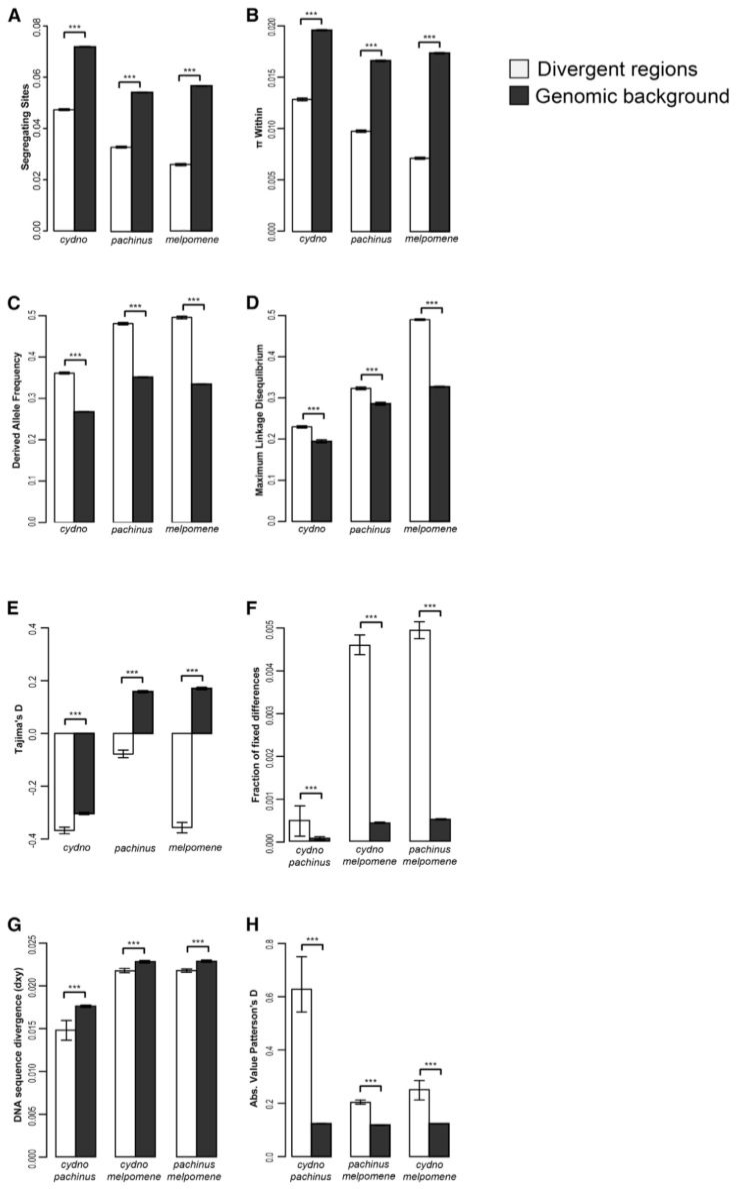
Divergent Regions of the Genome Exhibit Signatures of Selection and Adaptive Introgression Each panel shows the mean values of population genetic statistics inside divergent regions (white bars) versus the genomic background (gray bars). Segregating site density (A), π within species (B), derived allele frequency (C), maximum linkage disequilibrium (D), Tajima’s D (E), fraction of fixed differences between species (F), mean pairwise sequence divergence between species (*d_XY_*) (G), and absolute value of Patterson’s D statistic for the four taxon ordering: *H. cydno*, *H. pachinus*, *H. melpomene*, outgroup (*H. hecale* and *H. ismenius*) (H). Error bars (indicating 95% confidence intervals) and p values are based on bootstrap resampling. ***p < 0.0001.

To test these hypotheses, we scanned the genome with multiple population genetic statistics and then compared divergent regions to the rest of the genome. This analysis revealed multiple, classic signatures of divergent selection as well as evidence for long-term purifying selection. For instance, divergent regions displayed (1) reduced polymorphism (Figures 6A and 6B), (2) increased derived allele frequency (Figure 6C), (3) increased linkage disequilibrium (Figure 6D), and (4) negative Tajima’s D values (Figure 6E). Furthermore, consistent with a history of selective constraint following initial divergent selection, divergent regions were highly enriched for fixed differences between species (Figure 6F) yet showed reduced total sequence divergence (*d_XY_*) between species (Figure 6G), the latter being a classic signature of purifying selection (Haddrill et al., 2005; Halligan and Keightley, 2006; Marais et al., 2005; Parsch, 2003).

Finally, we wanted to determine the source of genetic variation contributing to divergence. Previous work has shown a signature of shared ancestry among *Heliconius* species around wing-patterning loci (Heliconius Genome Consortium, 2012; Pardo-Diaz et al., 2012; Smith and Kronforst, 2013), suggestive of a role for introgression in the evolution of mimicry. Given the amount of hybridization among these taxa, it is possible that interspecific gene flow may have played a more general role in facilitating adaptation. To test this possibility, we scanned the genome using Patterson’s D (Durand et al., 2011), a measure of shared ancestry, and then compared divergent regions to the rest of the genome. We found that divergent genome regions had more extreme values of *D*, compared to the rest of the genome (Figure 6H), and this pattern remained even after excluding divergence associated with mimicry loci (permutation test, p < 0.001). This introgression is likely to be adaptive because the signal of shared ancestry is enriched in these highly differentiated regions of the genome that also have multiple signatures of selection. Hence, adaptive introgression appears to be pervasive among hybridizing *Heliconius* species, potentially influencing many aspects of their biology.

### Conclusions

The study of speciation is inherently challenging because it generally involves inferring a piecemeal process of divergence after reproductive isolation is complete. Systems such as *Heliconius* permit direct investigation of the genetic changes associated with speciation because species that are phenotypically well differentiated, and often sympatric, continue to hybridize, reducing divergence at neutral sites. We validated this basic expectation of divergence with gene flow and then used the resultant heterogeneity in genomic divergence to characterize the shape and depth of the species boundary as a function of divergent selection, phylogenetic distance, and hybridization. Our results provide unique insights into (1) what defines genomic regions of divergence associated with speciation, (2) how divergence evolves over time, (3) what the targets of selection are at the genetic level, and (4) the repeatability of this process. Beyond that, our work reveals important, creative roles for both selection and introgression in the origin of species. It is quite possible that this combined action of gene flow and selection may have a more general role in driving instances of rapid diversification (Seehausen, 2004). In addition, these results help elucidate the relative roles of divergent selection, divergence hitchhiking, and genome hitchhiking during the process of speciation with gene flow. Specifically, our data point to an essential role for divergent selection in initiating speciation, and we also see signs consistent with genome hitchhiking later in the process. In contrast, the role of divergence hitchhiking appears to be modest relative to these other two processes. These empirical results agree well with recent simulations in which all three processes are allowed to operate (Feder et al., 2012b; Flaxman et al., 2013). Ongoing work in this and a variety of other biological systems (Hendry et al., 2009; Kitano et al., 2009; Martin et al., 2013; McKinnon and Rundle, 2002; Michel et al., 2010; Nosil et al., 2012a, 2012b) will help expand on the generality of these results.

## EXPERIMENTAL PROCEDURES

For more information, see Supplemental Experimental Procedures.

### Samples

We collected 32 samples from 13 locations across Costa Rica (Table S1) and sequenced each to an average depth of 16× coverage using an Illumina Hi-Seq 2000 (2 × 100 paired-end sequencing). These data were aligned to the Hmel 1.1 reference genome (Heliconius Genome Consortium, 2012) using Stampy (Lunter and Goodson, 2011) and SNPs were called simultaneously for all samples using the multiallelic calling function in GATK version 1.5 (DePristo et al., 2011; McKenna et al., 2010). The final data set consisted of 33,061,085 SNPs, with 97% of these sites covered in each sample (Table S2).

### Genome-wide Demographic Inference

Coalescent simulations, implemented in IMa2 (Hey, 2010; Nielsen and Wakeley, 2001), were used to generate neutral estimates of migration (*2Nm*), effective population size (θ), and divergence times (*t*μ;TMRCA). Ten 10 kbp windows were drawn randomly from each chromosome, and each window was phased using BEAGLE version 3.3.2 (Browning and Browning, 2007). The phased SNPs were converted to FASTA formatted haplotypes, and the longest nonrecombining block within each window was identified with IMgc (Woerner et al., 2007). Each of the resulting ten, 21 locus (representing each chromosome) data sets was analyzed in IMa2. Results are summarized across the ten data sets in Figure 1C, Table S3, and Table S7.

### Simulations

Gene trees were simulated under a neutral model using Hudson’s program *ms* (Hudson, 2002). The full migration model, with population size changes, was modeled as follows: ms 60 10000 −t 34.6 −I 3 20 20 20 −max 11.53 11.53 0 × 12.56 0 4.89 × −n 1 0.35 −n 2 1.59 −n 3 0.22 −ej 0.761 3 2 −en 0.761 2 0.035 −ej 2.48 2 1 −en 2.48 1 1. Coalescent trees without migration were simulated using the following command line: ms 60 10000 −t 34.6 −I 3 20 20 20 −n 1 0.35 −n 2 1.59 −n 3 0.22 −ej 0.761 3 2 −en 0.761 2 0.035 −ej 2.48 2 1 −en 2.48 1 1. Sixty 5 kbp DNA segments were then generated for each of the coalescent gene trees using Seq-Gen (Rambaut and Grassly, 1997) and used to determine the neutral distribution of F_ST_ for each comparison using Arlequin 3.5.1.3 (Excoffier and Lischer, 2010). F_ST_ distributions under models with and without migration were then compared to our empirical distributions (Figure S1).

### Identifying Divergent Genomic Regions

Every scaffold was divided into 5 kbp windows and F_ST_ values were calculated for each window in three pairwise comparisons: *H. cydno*-*H. pachinus*, *H. cydno*-*H. melpomene*, and *H. pachinus*-*H. melpomene*. To identify a common scale across which to compare genomic divergence, and to reduce the statistical nonindependence of F_ST_ comparisons for 5 kbp windows, we estimated empirical significance thresholds and linked adjacent windows that exhibited elevated differentiation (Figure S2). Windows with F_ST_ values greater than the 95th percentile (F_ST_ ≥ 0.598) were treated as highly divergent windows. For each pair of consecutive, though not necessarily adjacent, highly divergent windows, all the enclosed windows were classified as divergent if none of their F_ST_ values fell below the 75th percentile (F_ST_ ≥ 0.325).

### Population Genomics

For most of our analyses, we grouped samples by species, *H. cydno*, *H. pachinus*, and *H. melpomene*, except for those presented in Figure 5A, for which we separated *H. melpomene* samples into east and west collecting locations. We took the union of all divergent regions between the species pairs *H. cydno*-*H. pachinus*, *H. cydno*-*H. melpomene*, and *H. pachinus*-*H. melpomene* as a combined set, which was then compared to the remaining portion of the genomefor a variety of population genetic statistics (Figure 6). This set consisted of 941 genomic regions, containing 6,637 windows, spanning 32,983,224 bp of the genome (14.6% of the mapped chromosomes). The 97.5 and 2.5 percentile confidence intervals around the mean values were computed by bootstrap resampling from the entire set of windows 10,000 times. p values were estimated by bootstrap resampling and were adjusted to control for multiple tests (Benjamini and Hochberg, 1995). Pairwise linkage disequilibrium (LD) was calculated as the squared correlation coefficient (r^2^) between allele counts observed at two SNPs using the VCF tools software package (Danecek et al., 2011). This approach is computationally feasible for large data sets since it does not require haplotype reconstruction, but it provides only an approximation of the true LD (Rogers and Huff, 2009). Derived allele frequency and Patterson’s D both require identifying ancestral and derived alleles, which we did using *H. ismenius* and *H. hecale* as a combined outgroup.

### Clustering Analysis

To test if the counts of divergent regions were overrepresented or underrepresented on any chromosome in the *H. cydno*-*H. pachinus* comparison, we used a Monte-Carlo-simulated nonparametric paired Wilcoxon test (Z = −1.949, p = 0.05). The probability of observing regions of high divergence between *H. cydno* and *H. pachinus* on a chromosome containing a known color-pattern locus (chr1, chr10, chr15, chr18) was estimated using Fisher’s exact test (p < 0.01). Equivalent tests for *H. cydno*-*H. melpomene* and *H. pachinus*-*H. melpomene* were performed using the nonparametric simulated paired Wilcoxon test, as above (all *Z* ≤ −5.06, all p > 0.61). To test for enrichment of divergent regions on color-pattern chromosomes, we tested a contingency table of regions on color pattern chromosomes versus not on these chromosomes, normalized by chromosome length (Fisher’s exact tests, p > 0.538 in both cases).

### GO Term Enrichment Analysis

Gene sequences were extracted from Hmel1.1 and annotated using FlyBase and GO Elite. We combined permuted probabilities from the merged GO Elite analysis for the three interspecific comparisons using Fisher’s method and then adjusted the tests for multiple comparisons based on the total number of genes in the comparison set, multiplied by 3 to further correct for the three nonindependent comparisons (Table S6).

## Supplementary Material



## Figures and Tables

**Table 1:** Dynamics of Genome Divergence across the Heliconius Phylogeny

Species Pairing	No. of Divergent Regions	Cumulative Region Size (bp)	Average Region Size (bp)
*cydno*, *pachinus*	12	165,000	13,750
*cydno*, *melpomene*	688	18,949,219	27,542
*pachinus*, *melpomene*	933	32,615,794	34,958
