# Supplementary material for: Hybridization Reveals the Evolving Genomic Architecture of Speciation
Source: Cell Rep. Author manuscript; Available in PMC 2015 Apr 7. (PMC4388300; doi:10.1016/j.celrep.2013.09.042)
Supplement: Supplementary file 1 [file NIHMS598388-supplement-supplement_1.pdf]

Supplemental Information for:

Hybridization reveals the evolving genomic architecture of speciation

Marcus R. Kronforst, Matthew E. B. Hansen, Nicholas G. Crawford, Jason R. Gallant, Wei Zhang, Rob J. Kulathinal, Durrell D. Kapan & Sean P. Mullen

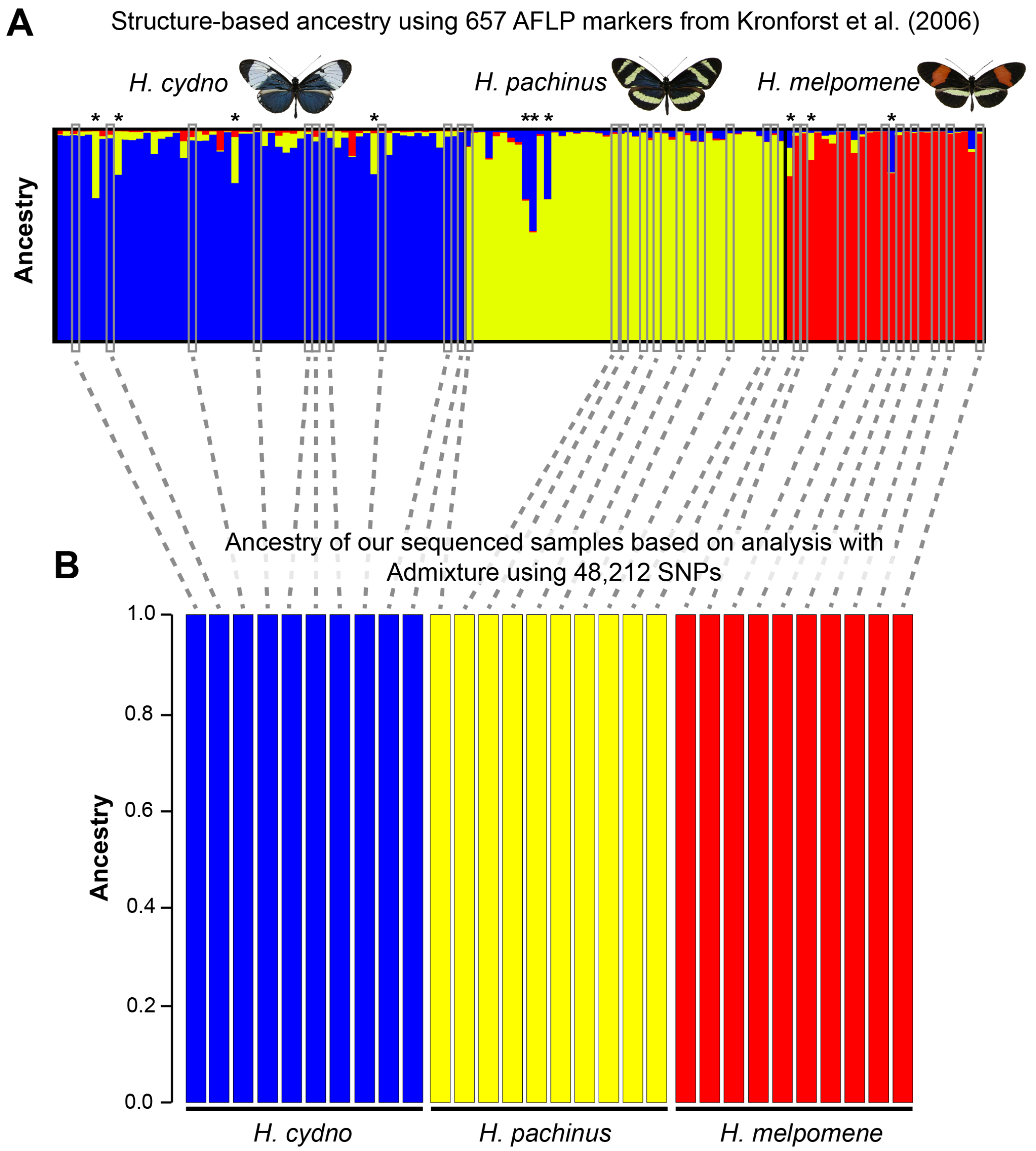

Figure S1. A) Previous work detected widespread admixture among *H. cydno*, *H. pachinus* and *H. melpomene* in Costa Rica (Kronforst et al., 2006b). We specifically selected samples from this set that did not show evidence of recent admixture so as to not bias our estimates of divergence and gene flow among species. (B) Subsequent analysis based on 48K of our genotyped SNPs verified that sequenced samples did not have recent hybrid ancestry. This analysis, run with the program ADMIXTURE 1.2, was based on a small subset of our total SNP dataset because we sampled polymorphisms with a minimum spacing of 5 kbp, a value set by the extent of LD in the genome (Figure S6).

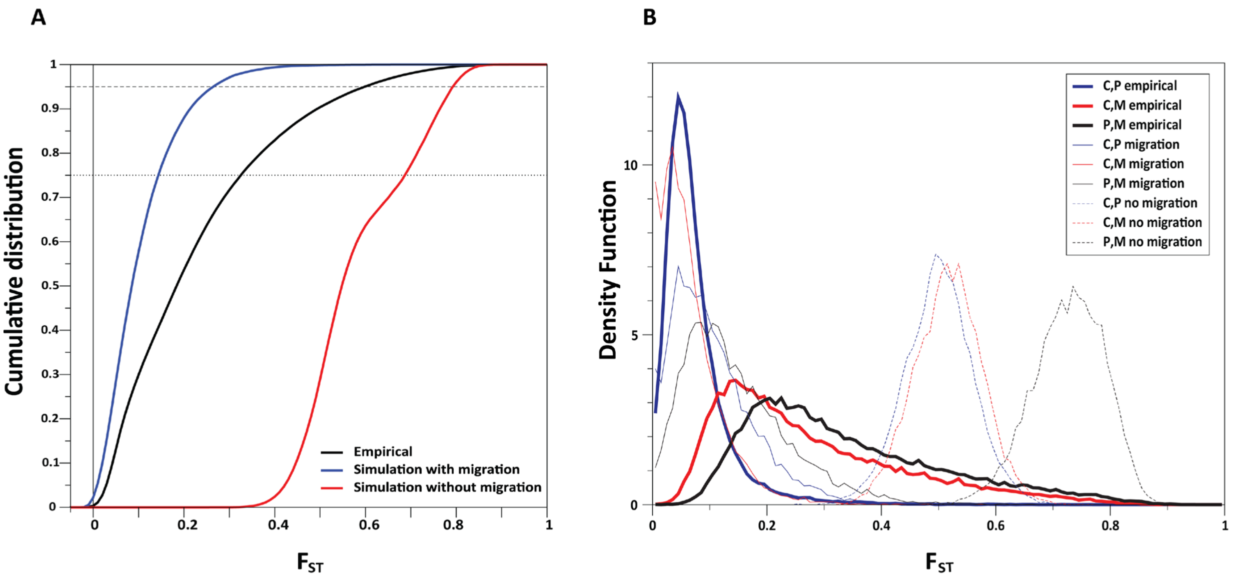

Figure S2. A) Cumulative F_{ST} distribution for 1) empirical data (blue), 2) simulated data under a Fisher-Wright neutral model with migration (black), and 3) simulated data under a neutral model without migration (red). Dashed lines represent the 95% (-) and 75% (˙) distribution thresholds, respectively. B) Density function of 5 kbp window F_{ST} values for each pairwise species comparison: empirical values are shown in solid (bold) lines, simulated values under a Fisher-wright neutral model with migration are also shown as solid (unbolded) lines, simulated values under a Fisher-Wright neutral model without migration are displayed as dashed lines. Iterative experimental manipulations of simulation parameters indicate that the left-shifted F_{ST} values observed for our *H. cydno-H. melpomene* data simulated under a neutral no migration model result from the large N_e of *H. cydno* relative to *H. melpomene*.

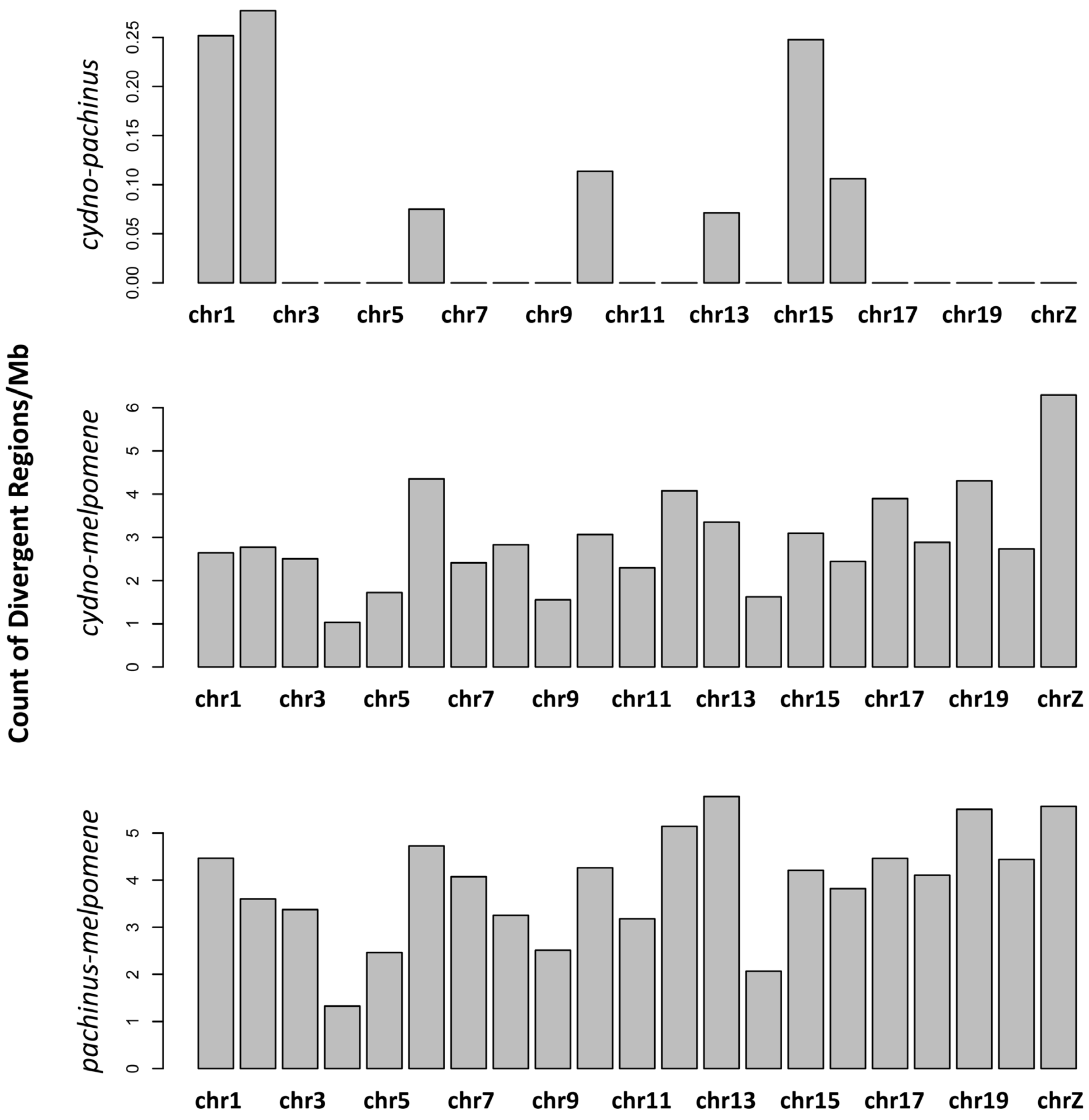

Figure S3. Clustering of highly divergent regions by chromosome for 1) *cydno-pachinus* (top), 2) *cydno-melpomene* (middle), and 3) *pachinus-melpomene* (bottom).

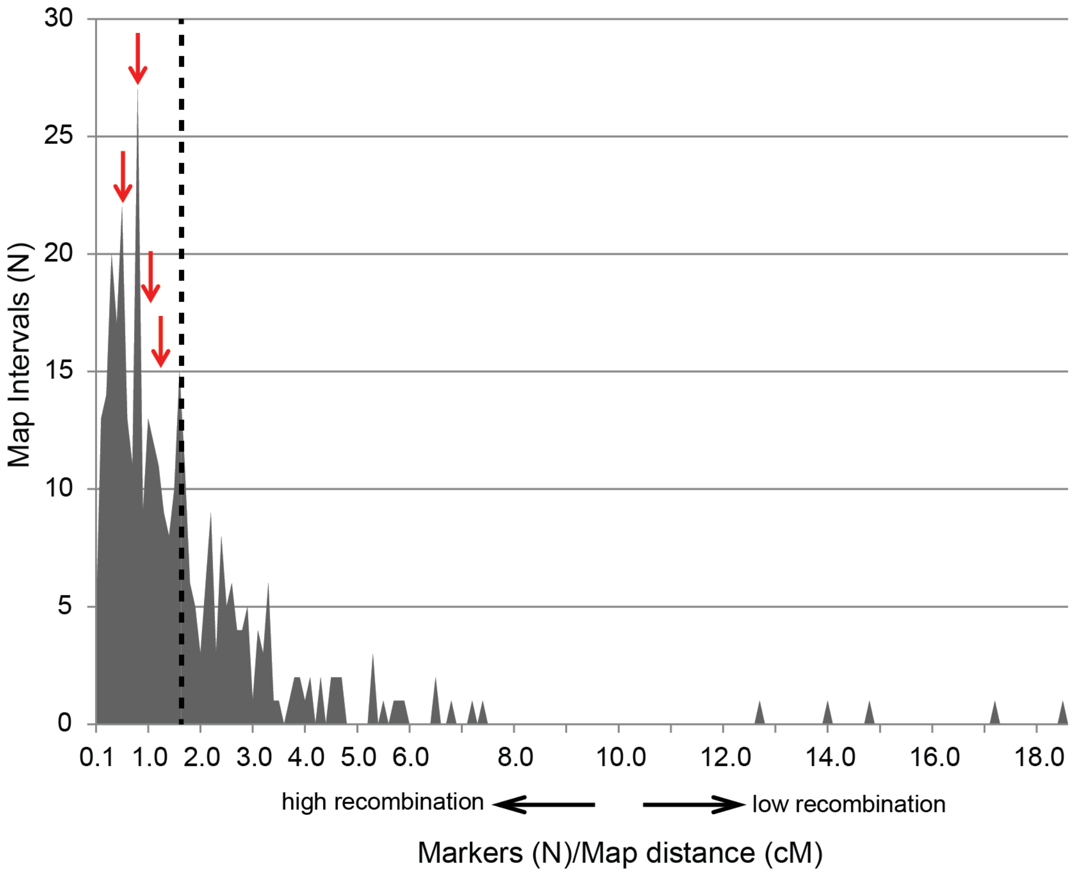

Figure S4. Linkage mapping reveals that *Heliconius* mimicry loci are not in regions of reduced recombination. We quantified recombination across the genome based on our full genome linkage map (Kronforst et al., 2006a; Kronforst et al., 2006c). Areas of reduced recombination will emerge as clusters of tightly-linked markers, quantified here as a high marker/cM ratio (x-axis). The mean marker/cM ratio across the genome is 1.7 (dashed line) and all four of the major wing pattern mimicry loci (red arrows) are located in regions with lower values, indicating high levels of recombination. Since the mimicry loci stand out as the most strongly divergent regions in our analyses, this suggests that observed signatures of divergence and selection are not a by-product of reduced recombination.

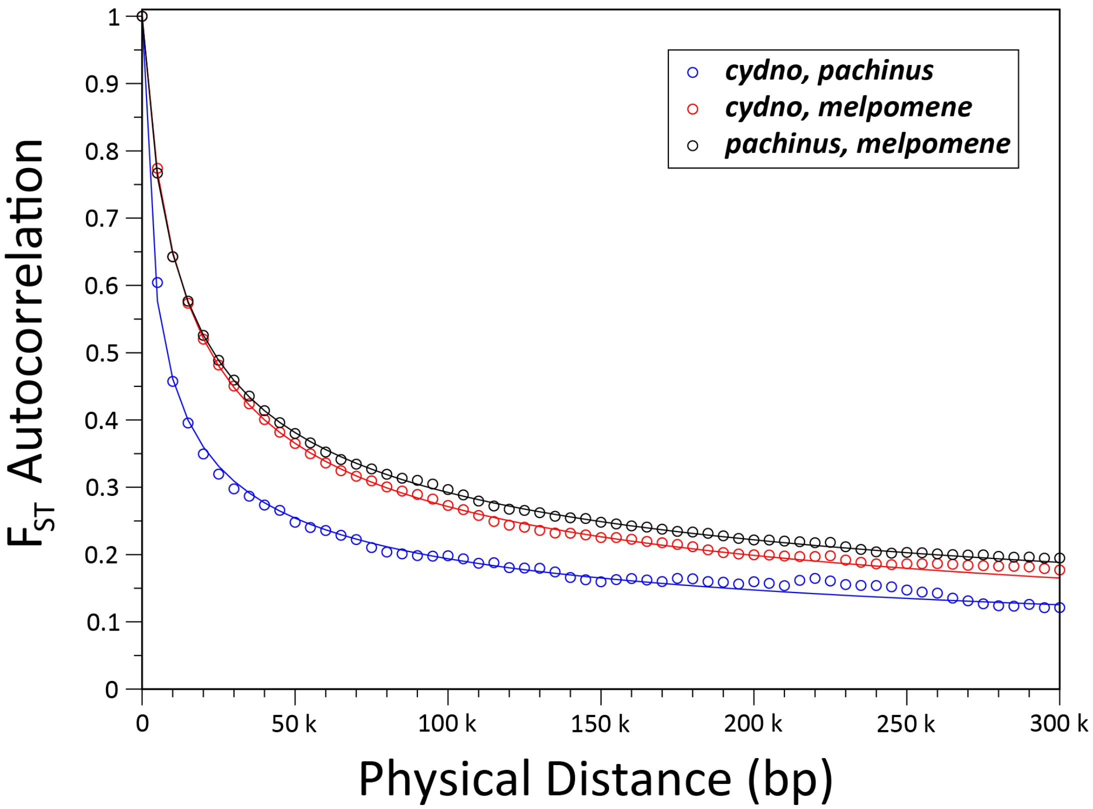

Figure S5. F_{ST} autocorrelation among sites fit to an exponentially declining power law (exponent -0.4 to -0.47 across the three comparisons) enforcing the ACF constraint y(x=0) = 1. For all three pairwise ACFs the correlation coefficient (r) exceeds 0.998.

Figure S6. Pairwise Linkage Disequilibrium (LD) measured as the squared correlation coefficient (r^2) between two SNPS for each species within (red) and outside (black) divergent genomic regions.

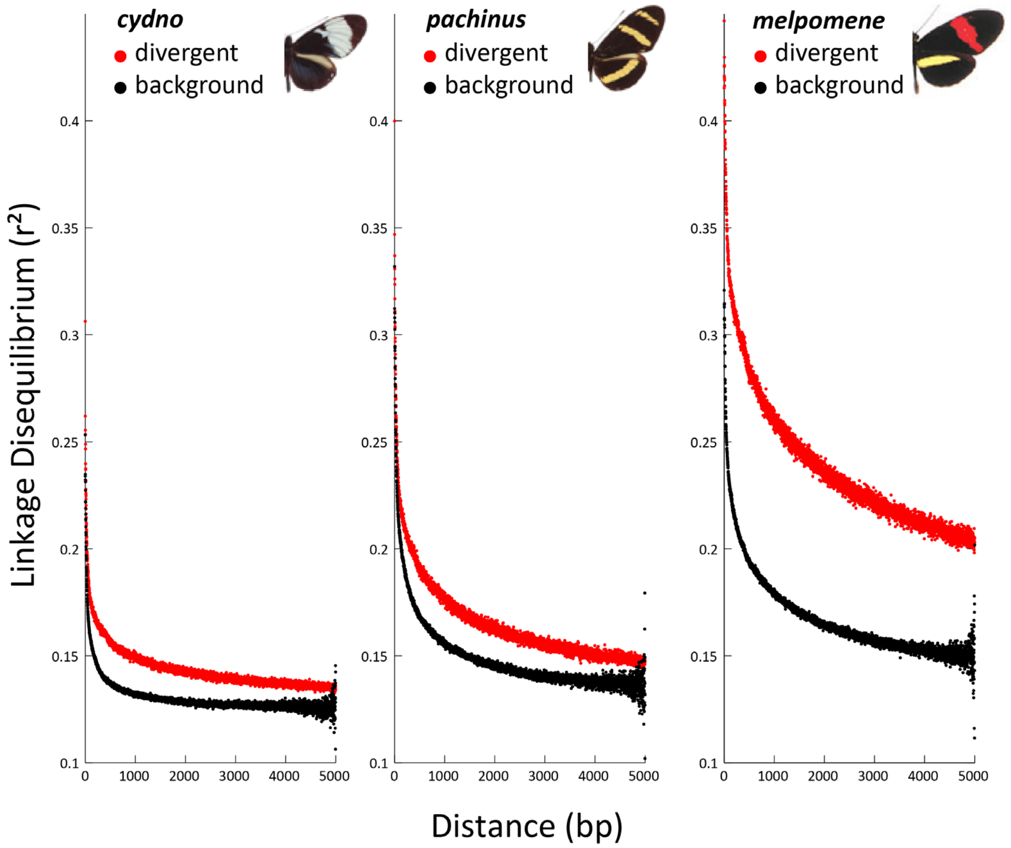

Figure S6. Pairwise Linkage Disequilibrium (LD) measured as the squared correlation coefficient (r^2) between two SNPS for each species within (red) and outside (black) divergent genomic regions.

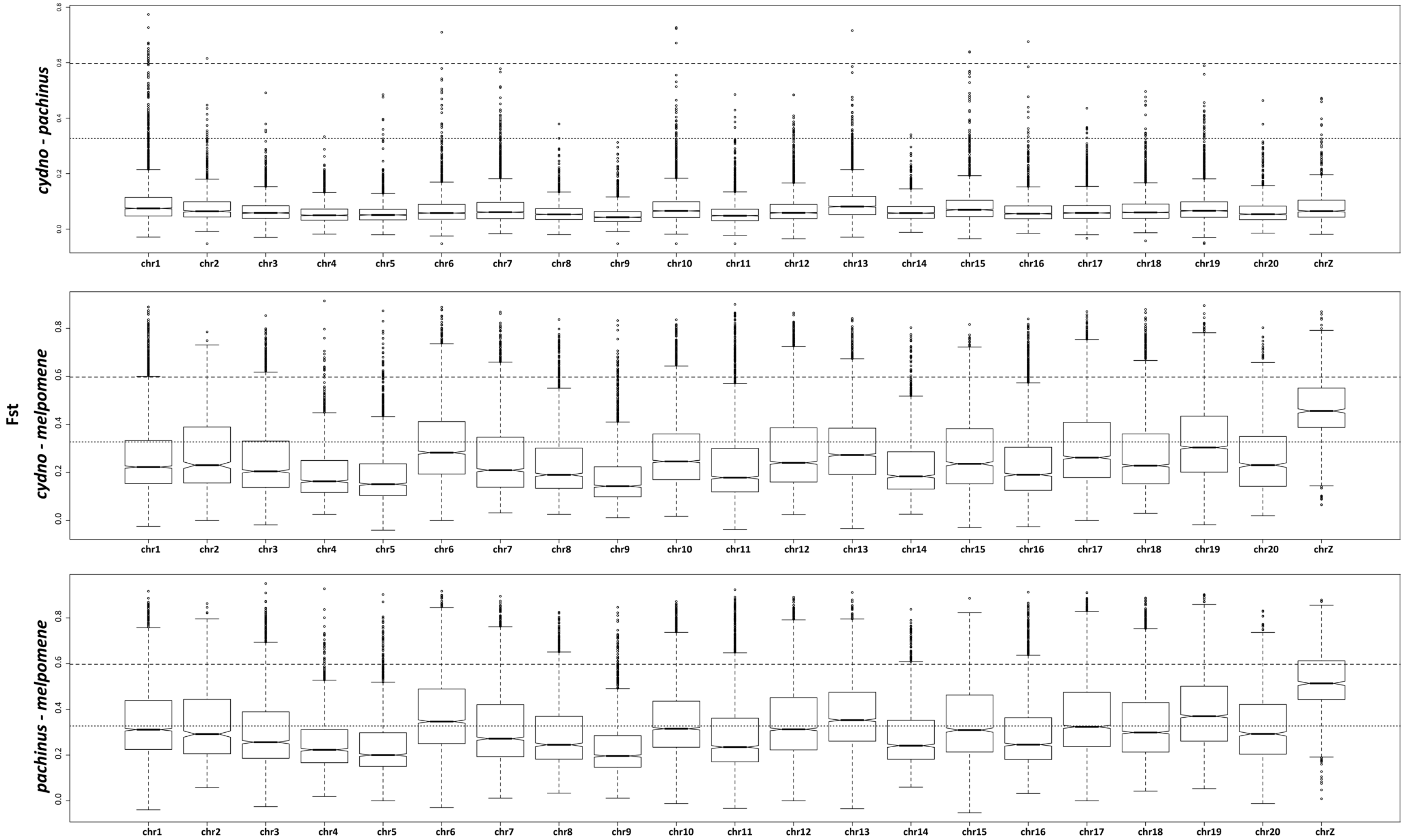

Figure S7. Pairwise F_{ST} represented as boxplots with whiskers between 1) *cydno-pachnius* (top), 2) *cydno-melpomene* (middle), and 3) *pachinus-melpomene* (bottom) for all 21 chromosomal regions highlighting elevated divergence on the Z-chromosome in comparisons with *H. melpomene*.

Table S1. Sample collection information.

Sample	Species	sex	location	Coast	Lat.	Long.
c511	*cydno galanthus*	female	Cariblanco	Caribbean	10° 16' N	84° 11' W
c512	*cydno galanthus*	male	PN Hitoy Cerere	Caribbean	9° 40' N	83° 2' W
c513	*cydno galanthus*	male	La Selva Biological Station	Caribbean	10° 26' N	83° 59' W
c514	*cydno galanthus*	female	Vesta	Caribbean	9° 43' N	83° 3' W
c515	*cydno galanthus*	female	Guacimo	Caribbean	10° 13' N	83° 41' W
c563	*cydno galanthus*	male	Guapiles	Caribbean	10° 13' N	83° 47' W
c614	*cydno galanthus*	male	Vesta	Caribbean	9° 43' N	83° 3' W
c630	*cydno galanthus*	female	La Selva Biological Station	Caribbean	10° 26' N	83° 59' W
c639	*cydno galanthus*	male	Guacimo	Caribbean	10° 13' N	83° 41' W
c640	*cydno galanthus*	female	Vesta	Caribbean	9° 43' N	83° 3' W
h665	*hecale*	male	La Selva Biological Station	Caribbean	10° 26' N	83° 59' W
i02_210	*ismenius*	male	PN Manuel Antonio	Pacific	9° 24' N	84° 10' W
m523	*melpomene rosina*	male	Vesta	Caribbean	9° 43' N	83° 3' W
m589	*melpomene rosina*	male	Selva Bananito	Caribbean	9° 52' N	83° 0' W

m676	*melpomene rosina*	male	Vesta	Caribbean	9° 43' N	83° 3' W
m682	*melpomene rosina*	male	La Selva Biological Station	Caribbean	10° 26' N	83° 59' W
m687	*melpomene rosina*	female	La Selva Biological Station	Caribbean	10° 26' N	83° 59' W
m524	*melpomene rosina*	female	Puriscal	Pacific	9° 51' 0N	84° 19' W
m525	*melpomene rosina*	male	Sirena Biological Station	Pacific	8° 28' N	83° 35' W
m675	*melpomene rosina*	male	PN Manuel Antonio	Pacific	9° 24' N	84° 10' W
m683	*melpomene rosina*	male	PN Manuel Antonio	Pacific	9° 24' N	84° 10' W
m689	*melpomene rosina*	female	Puriscal	Pacific	9° 51' 0N	84° 19' W
p516	*pachinus*	female	Puriscal	Pacific	9° 51' 0N	84° 19' W
p517	*pachinus*	male	Puriscal	Pacific	9° 51' 0N	84° 19' W
p518	*pachinus*	male	PN Manuel Antonio	Pacific	9° 24' N	84° 10' W
p519	*pachinus*	female	Sirena Biological Station	Pacific	8° 28' N	83° 35' W
p520	*pachinus*	male	Sirena Biological Station	Pacific	8° 28' N	83° 35' W
p591	*pachinus*	male	Colon	Pacific	9° 55' N	84° 15' W
p596	*pachinus*	male	PN Carara	Pacific	9° 47' N	84° 36' W
p690	*pachinus*	female	Puriscal	Pacific	9° 51' 0N	84° 19' W

p694	*pachinus*	female	PN Manuel Antonio	Pacific	9° 24' N	84° 10' W
p696	*pachinus*	female	Sirena Biological Station	Pacific	8° 28' N	83° 35' W

Table S2. Sequence coverage and SNP scoring statistics.

Sample	Sequencing coverage (X)	Effective Coverage (X)	Polymorphic Sites w/ Genotypes	Total SNPs/sample
c511	15.98	14.57	32,330,521	7,834,670
c512	16.13	14.67	32,339,964	7,880,908
c513	16.19	14.83	32,323,062	7,865,793
c514	16.12	14.73	32,302,561	7,805,667
c515	16.06	14.70	32,271,197	7,781,108
c563	15.14	13.83	32,242,026	7,831,760
c614	16.07	14.81	32,346,747	7,885,840
c630	15.24	13.83	32,258,915	7,787,160
c639	16.13	14.84	32,274,434	7,841,661
c640	16.15	14.82	32,276,539	7,799,394
h665	16.04	14.13	31,263,335	9,026,075
i02-210	15.90	13.61	30,292,045	8,522,654
m523	16.26	14.94	32,351,936	5,732,343
m524	16.06	15.03	32,386,948	5,838,627
m525	16.20	14.58	32,430,751	5,848,993
m589	16.11	14.92	32,299,253	5,704,349
m675	16.06	15.07	32,399,303	5,835,224
m676	14.95	13.63	32,218,837	5,631,994
m682	16.03	14.96	32,302,390	5,646,811
m683	15.01	14.19	32,346,131	5,808,734

m687	16.11	15.06	32,298,593	5,637,142
m689	15.97	14.71	32,398,193	5,835,221
p516	16.64	14.94	32,200,990	7,489,584
p517	16.63	15.26	32,220,174	7,516,119
p518	16.63	15.23	32,221,498	7,517,439
p519	16.62	15.15	32,180,303	7,454,787
p520	15.02	13.78	32,145,249	7,512,737
p591	15.12	13.84	32,148,217	7,518,538
p596	15.21	13.72	32,121,475	7,497,475
p690	16.63	14.85	32,143,341	7,439,867
p694	16.64	15.06	32,210,561	7,486,019
p696	16.66	15.19	32,214,529	7,478,731
**mean**	**15.99**	**14.61**	**32,180,001**	**7,134,170**

Table S3. Summary of demographic parameters inferred using IMa2. These are mean and 95% CI based on 10 independent runs of IMa2, using different loci in each run. Parameter estimates and 95% CI for each IMa2 run are shown in Table S7.

**Population sizes**	**Mean**	**CI**
cydno (C)	1.81E+06	3.53E+05
pachinus (P)	2.55E+05	4.85E+04
melpomene (M)	5.13E+05	4.56E+05
C, P MRCA	4.04E+04	2.43E+04
C, P, M MRCA	1.13E+06	6.03E+05
**Divergence times**		
C vs. P	4.29E+05	7.36E+04
M vs. C, P MRCA	1.36E+06	2.08E+05
**Migration Rates**		
C into P	0.546	0.218
P into C	9.905	2.374
M into C	0.018	0.033
C into M	0.054	0.056
M into P	0.000	NA
P into M	0.072	0.055
M into C, P MRCA	0.019	0.023
C, P MRCA into M	1.989	1.192

Table S4. Genome sequence data suggest very few potential inversions in the locations of the 12 genomic regions that are divergent between *H. cydno* and *H. pachinus*. ‘Yes’ and ‘No’ below refer to whether an inversion is indicated for each sample in the genomic intervals displayed in Figure 3.

**Sample**	**Chr1**	**Chr2**	**Chr6**	**Chr10a**	**Chr10b**	**Chr13**	**Chr15**	**Chr16**	**Chr18**
c511	No	Yes	No	No	No	No	No	No	No
c512	No	No	No	No	No	No	No	No	No
c513	No	No	No	No	No	No	No	No	No
c514	No	No	No	No	No	No	No	No	No
c515	Yes	Yes	No	No	No	No	No	No	No
c563	No	No	No	No	No	No	No	No	No
c614	No	Yes	No	No	No	No	No	No	No
c630	No	No	No	Yes	No	No	No	No	No
c639	No	No	No	No	No	No	No	No	No
c640	No	No	No	No	No	No	No	No	No
h665	No	No	No	No	No	No	No	No	No
i02_210	No	No	No	No	No	No	No	No	No
m523	No	No	No	No	No	No	No	No	No
m589	No	No	No	No	No	No	No	No	No
m676	No	No	No	No	Yes	No	No	No	No
m682	No	No	No	No	No	No	No	No	No
m687	No	No	No	No	No	No	No	No	No
m524	No	No	No	No	No	No	No	No	No

m525	No	No	No	No	No	No	No	No	No
m675	No	No	No	No	No	No	No	No	No
m683	No	No	No	No	No	No	No	No	No
m689	No	No	No	No	No	No	No	No	No
p516	No	No	No	No	No	No	No	No	No
p517	No	No	No	No	No	No	No	No	No
p518	No	No	No	No	No	No	No	No	No
p519	No	No	No	No	No	No	No	No	No
p520	No	Yes	No	No	No	No	No	No	No
p591	Yes	No	No	No	No	No	No	No	No
p596	No	No	No	No	No	No	No	No	No
p690	No	No	No	No	No	No	No	No	No
p694	No	No	No	No	No	No	No	No	No
p696	No	No	No	No	No	No	No	No	No

Table S5. Estimates of fixed differences between populations/species. For each pairing, five individuals were sampled at random without replacement from *cydno* and/or *pachinus*, and all five individuals were used for *melpomene* East and/or *melpomene* West. The number of sites with fixed differences were then computed. This was done 20 times, with different groupings of five individuals taken each time for *cydno* and *pachinus*. Only sites with complete data (genotypes for all 10 individuals in the pairing) were used.

Group 1	Group 2	Mean Fraction of Fixed Differences	SD
melpomene East	*melpomene* West	4.24E-07	NA[1]
cydno	*pachinus*	1.09E-04	1.41E-05
cydno	*melpomene* East	9.20E-03	1.43E-04
cydno	*melpomene* West	9.55E-03	1.02E-04
pachinus	*melpomene* East	1.34E-02	2.35E-04
pachinus	*melpomene* West	1.39E-02	2.65E-04

[1] The *melpomene* East vs. *melpomene* West pairing involved no resampling because there are a total of five samples in each group.

Table S6. GO term enrichment analysis, comparing divergent genome regions to the entire genome.

Ontology ID	Ontology term	Ontology type	Adjusted P
GO:0000339	RNA cap binding	molecular function	5.20E-11
GO:0046914	transition metal ion binding	molecular function	6.57E-11
GO:0044427	chromosomal part	cellular component	1.36E-07
GO:0032312	regulation of ARF GTPase activity	biological process	7.19E-07
GO:0051119	sugar transmembrane transporter activity	molecular function	1.95E-06
GO:0008513	secondary active organic cation transmembrane transporter activity	molecular function	2.85E-06
GO:0003824	catalytic activity	molecular function	1.96E-05
GO:0060250	germ-line stem-cell niche homeostasis	biological process	1.13E-04
GO:0008306	associative learning	biological process	8.87E-04
GO:0045466	R7 cell differentiation	biological process	4.31E-03
GO:0043035	chromatin insulator sequence binding	molecular function	6.74E-03
GO:0050803	regulation of synapse structure and activity	biological process	7.03E-03
GO:0051783	regulation of nuclear division	biological process	8.16E-03
GO:0005700	polytene chromosome	cellular component	8.92E-03
GO:0001076	RNA polymerase II transcription factor binding transcription factor activity	molecular function	1.10E-02
GO:0008060	ARF GTPase activator activity	molecular function	1.10E-02
GO:0040020	regulation of meiosis	biological process	1.52E-02
GO:0030703	eggshell formation	biological process	2.38E-02

GO:0045595	regulation of cell differentiation	biological process	2.90E-02
GO:0045742	positive regulation of epidermal growth factor receptor signaling pathway	biological process	3.04E-02
GO:0005049	nuclear export signal receptor activity	molecular function	3.39E-02
GO:0048598	embryonic morphogenesis	biological process	3.81E-02
GO:0030163	protein catabolic process	biological process	4.10E-02
GO:0044260	cellular macromolecule metabolic process	biological process	4.15E-02
GO:0045010	actin nucleation	biological process	4.42E-02
GO:0030659	cytoplasmic vesicle membrane	cellular component	4.59E-02

Table S7. Estimates and 95% CI for each parameter from the 10 IMa2 runs.

See Kronforst et al Table S7.xlsx.

Supplemental Experimental Procedures

Samples

We collected samples from 13 locations across Costa Rica (Table S1). Samples were collected in the field as adults, euthanized, and then wings were separated and placed in glassine envelopes while the bodies were stored in 95% ethanol. For each specimen, genomic DNA was extracted from a portion of thoracic tissue using a DNeasy Blood & Tissue Kit (Qiagen).

Sequencing

A custom Illumina sequencing library with a 500 bp insert was prepared for each sample and sequenced to an average depth of 16X coverage using an Illumina Hi-Seq 2000 (2 × 100 paired-end sequencing). Library preparation and sequencing were performed at BGI. In total, we generated 182.7 Gbp of data across the 32 libraries. Raw reads were preprocessed to trim adaptor sequences and remove low quality reads. These data were subsequently aligned to the Hmel 1.1 reference genome (Heliconius Genome Consortium, 2012) using Stampy (Lunter and Goodson, 2011). SNPs were called simultaneously for all samples using the multi-allelic calling function in GATK version 1.5 (DePristo et al., 2011; McKenna et al., 2010). Positions with a total SNP quality less than 40 were filtered from subsequent analyses. Females have only one Z chromosome but due to sequencing errors, our SNP calling pipeline occasionally, but rarely, scored females as heterozygotes for Z-linked SNPs. However, GATK gives a probability score to each allele and we found that these were not the same in the case of female Z-linked heterozygous sites, likely as a result one allele being due to sequencing error. We edited these sites to assign them single alleles by retaining the single, highest probability nucleotide at each site. The final dataset consisted of 33,061,085 SNPs, with 97% of these sites covered in each sample (Table S2).

Phylogeny

To calculate a genome wide species tree, 4,051 non-overlapping 50 kbp windows were drawn from the multi-allelic VCF file. Scaffolds less than 50 kbp were excluded. Windows were centered within scaffolds (e.g., for a scaffold of 60 kbp the window started at 5 kbp and ended at 55 kbp). Trees were calculated for each window with PhyML v3.0_360-500M using an HKY DNA substitution model. The summary tree is shown in Figure 1A.

Genome-wide Demographic Inference

Coalescent simulations, implemented in IMa2 (Hey, 2010; Nielsen and Wakeley, 2001), were used to generate neutral estimates of migration (*2Nm*), effective population size (θ), and divergence times ($t\mu$;TMRCA). To generate phased haplotype input files for

IMa2, bi-allelic SNPs were called independently for each species using GATK with the same filtering parameters described above for the multi-allelic analyses. The resulting VCF files were fed through the GATK walker "ProduceBeagleInputWalker" to generate likelihood files in BEAGLE format. Ten 10 kbp windows were then drawn randomly from each chromosome, for a total 210 windows (2.1 Mbp of sequence), and each window was phased using the software program BEAGLE version 3.3.2 (Browning and Browning, 2007). The phased SNPs were converted to FASTA formatted haplotypes and the longest non-recombining block within each window was identified with IMgc (Woerner et al., 2007). Each of the resulting ten, 21 locus (representing each chromosome) datasets was analyzed in IMa2 under an HKY model of mutation, using 100 geometrically heated chains (0.99, 0.75), and a pre-defined species tree. After discarding the first 150K steps as burn-in, each simulation was allowed to proceed until the parameter estimates from the first and second half of the run converged. We then used 10K sampled coalescent genealogies to estimate population sizes, bi-directional migration rates, splitting times, and mutation scalars. Results are summarized across the ten data sets in Figure 1C and Table S3.

Simulations

To assess the interplay between selection, drift, and demographic history, 10,000 coalescent gene trees were simulated under a neutral model, within the species level phylogeny for our three focal taxa (*H. cydno*, *H. pachinus* & *H. melpomene*; Figure 1A), using Hudson's program ms (Hudson, 2002). Mean estimates of individual demographic parameters were obtained via 10 independent, replicate IMa2 analyses (see above), and used to parameterize neutral models with and without migration. The full migration model, with population size changes, was modeled as: ms 60 10000 -t 34.6 -I 3 20 20 20 -ma x 11.53 11.53 0 x 12.56 0 4.89 x -n 1 0.35 –n 2 1.59 –n 3 0.22 -ej 0.761 3 2 –en 0.761 2 0.035 -ej 2.48 2 1 –en 2.48 1 1. Coalescent trees without migration were simulated using the following command line: ms 60 10000 -t 34.6 -I 3 20 20 20 -n 1 0.35 –n 2 1.59 –n 3 0.22 -ej 0.761 3 2 –en 0.761 2 0.035 -ej 2.48 2 1 –en 2.48 1 1.

60 5-kbp DNA segments, corresponding to 10 sampled diploid genomes for each of the three focal species, were then generated for each of the 10,000 coalescent gene trees using Seq-Gen (Rambaut and Grassly, 1997), assuming an HKY model of molecular evolution. The resulting simulated DNA sequences were used to determine the neutral distribution of F_{ST} for each of the three pairwise comparisons by calculating divergence across 10,000 5-kbp windows using a custom Perl bioinformatics pipeline and the program Arlequin 3.5.1.3 (Excoffier and Lischer, 2010). F_{ST} distributions under models with and without migration were then compared to our empirical distributions (Figure S1).

Identifying Divergent Genomic Regions

Every scaffold was divided into 5 kbp tiling windows, with the last window on each scaffold taking up the remainder (window sizes less than 5 kbp). F_{ST} values were calculated for each window in the following pairwise comparisons *H. cydno-H. pachinus*, *H. cydno-H. melpomene*, and *H. pachinus-H. melpomene*. The F_{ST} autocorrelation function (ACF) was computed across the entire genome for each population pair. Each ACF, over the distance range of 0 – 3 Mbp, is well fit by a modified power law of the form $y(x) = a(x+b)^p$ with the parameter constraint y(0)=1, yielding two free fitting parameters, as shown in Figure S5. The correlation coefficient (r) of each fit exceeds 0.988. The best fit parameters (a,b,p) for the three population pairs are *H. cydno-H. pachinus*: (19.6, 1691.5, -0.401), *H. cydno-H. melpomene*: (62.6, 6650.8, -0.470), and *H. pachinus-H. melpomene*: (34.6, 5349.1, -0.413).

To identify a common scale across which to compare genomic divergence and to reduce the statistical non-independence of F_{ST} comparisons for 5 kbp windows, we estimated empirical significance thresholds and linked adjacent windows that exhibited elevated differentiation. To do this, all F_{ST} values from the three pairwise comparisons were combined and used to identify global 95th and 75th percentiles of 0.598 and 0.325, respectively (Figure S2). We also compared the observed F_{ST} distributions to distributions obtained from simulations with and without interspecific gene flow (Figure S2). For our analyses, it was essential to apply the same F_{ST} threshold across pairwise comparisons, although each comparison exhibits a different amount of divergence, because we were focused on identifying and then comparing the location and physical size of divergent segments. These comparisons are only possible if we identify a common cut-off across the combined F_{ST} distributions and this cut-off is held constant. Windows with F_{ST} values greater than the 95th percentile ($F_{ST} \geq 0.598$) were treated as highly divergent windows. By comparing observed and simulated F_{ST} distributions, we found that values above the 95th percentile represented outliers relative to simulations with interspecific gene flow but no selection, making this a robust and conservative cut-off. For each pair of consecutive, though not necessarily adjacent, highly divergent windows, all the enclosed windows were classified as divergent if none of their F_{ST} values fell below the 75th percentile ($F_{ST} \geq 0.325$). After one iteration, this resulted in a set of divergent regions, each composed of windows with F_{ST} values at or above the 75th percentile and bounded by windows at or above the 95th percentile. Windows separated by more than 5 kbp in the chromosomal coordinates were not treated as consecutive and divergent regions were not allowed to cross such gaps between windows.

Population Genomics

Population Statistics - For most of our analyses, we grouped samples by species, *H. cydno*, *H. pachinus*, and *H. melpomene*. However, in two analyses we examined how genome-wide divergence evolves over time, focusing on both the cumulative portion of the genome contained in divergent regions and mean d_{XY} (Figure 5A). For these analyses only, we separated *H. melpomene* samples into east and west collecting locations, to estimate within species divergence at t=0 (IMa2 analyses consistently yielded a *melpomene* East vs. *melpomene* West divergence time equal to the smallest sampled parameter value which is effectively zero).

For all our analyses, the following population genetic statistics were calculated over each window using the command line version of Arlequin 3.5.1.3: F_{ST}, segregating sites, π within species, d_{XY}, and Tajima's D. The data were treated as unphased genotypic data for every individual, except for the Z chromosome where the window sequence data was input as two haplotypes for each male and a single haplotype for each female.

Comparing Divergent Regions to the Genome - We took the union of all divergent regions between the species pairs *H. cydno*-*H. pachinus*, *H. cydno*-*H. melpomene*, and *H. pachinus*-*H. melpomene* as a combined set, which was then compared to the remaining portion of the genome for a variety of population genetic statistics. This set consisted of 941 genomic regions, containing 6,637 windows, spanning 32,983,224 bp of the genome (14.6% of the mapped chromosomes). Comparisons between divergent regions and the rest of the genome focused on within species statistics calculated across the union of all divergent regions. The 97.5 and 2.5 percentile confidence intervals around the mean values were computed by bootstrap resampling from the entire set of windows 10,000 times. *P*-values, comparing the difference between the means for within and outside divergent regions, were estimated by bootstrap resampling and were adjusted to control for multiple tests (Benjamini and Hochberg, 1995).

Linkage Disequilibrium - Pairwise Linkage Disequilibrium (LD), within and outside divergent genomic regions, was calculated as the squared correlation coefficient (r^2) between allele counts observed at two SNPs using the VCFtools software package (Danecek et al., 2011). This approach is computationally feasible for large data sets since it does not require haplotype reconstruction, but it provides only an approximation of the true LD (Rogers and Huff, 2009). A total of 6,000 5 kbp windows, representing highly divergent regions across all comparisons of our three focal species, were analyzed, and LD within these regions was compared to LD within 6,000 randomly sampled 5 kbp windows representing the genomic background. Pairwise linkage disequilibrium for divergent regions and background windows were separately averaged in 1bp bins for each species (Figure S6). Clear differences in pairwise LD can be observed between divergent and background comparisons within species, and also

between species. r^2 and log (distance) was linearly regressed for each comparison, and 95% confidence intervals were calculated by bootstrapping with 1000 replicates. Results of these comparisons are shown in Figure 6D.

Derived Allele Frequency and Patterson's D Statistics - The mean derived allele frequency and Patterson's D statistic were computed over each window. Both statistics require polarizing the allelic type into ancestral and derived. For this purpose, the *H. ismenius* sample and the *H. hecale* sample were, together, used as the outgroup. Only sites where the outgroup was fixed and had at least two acceptable allele calls were retained within each window. The mean derived allele frequency per species per window was computed as the mean frequency of non-ancestral alleles at sites that segregate within the population. For Patterson's D statistic, we further reduced the sites in each window to those that were strictly bi-allelic. Patterson's D was calculated in true phylogenetic order (*H. cydno*, *H. pachinus*, *H. melpomene*, outgroup; Figure 1A), thus testing the impact of gene-flow between *H. pachinus* and *H. melpomene* (ABBA) and *H. cydno* and *H. melpomene* (BABA). Patterson's D generates a single statistic across the three species, so in this case we compared its absolute value as a measure of increased bi-directional gene-flow amongst the three sets of divergent regions, *H. cydno-H. pachinus* regions, *H. pachinus-H. melpomene* regions and *H. cydno-H. melpomene* regions (Figure 6H).

Clustering Analysis

H. cydno – H. pachinus - To test if the counts of divergent regions were overrepresented or underrepresented on any chromosome (versus a null expectation based on chromosome length) we used a Monte-Carlo simulated non-parametric paired Wilcoxon test ($Z = -1.949$, $p=0.05$). The probability of observing regions of high divergence between *H. cydno* and *H. pachinus* on a chromosome containing a known color-pattern locus is given by a contingency table of color pattern chromosomes (chr1, chr10, chr15 & chr18) versus not. Since there were so few observations in this comparison, we only counted the numbers by chromosome and did not take into account chromosome size (Fisher's exact test for number of diverged regions in these two chromosome classes, simulated $p < 0.01$).

Comparisons with *H. melpomene* - Equivalent tests for *H. cydno – H. melpomene* and *H. pachinus – H. melpomene* were performed using the non-parametric simulated paired Wilcoxon test, as above. In all cases, the distribution of diverged regions did not differ from the random expectation based on placing the same number of regions on chromosomes in proportion to their size (all $Z <= -5.06$, all $p > 0.61$). To test for enrichment of divergent regions on color-pattern chromosomes versus non-color pattern chromosomes for both comparisons (*H. cydno – H. melpomene* and *H. pachinus – H.*

melpomene) we tested a contingency table of regions on color pattern chromosomes (chr1, chr10, chr15 & chr18) versus not on these chromosomes normalized by chromosome length. The observed number did not differ from the null hypothesis of no clustering (Fisher's exact tests, $p > 0.538$ in both cases).

Structural variant detection

We used pair-end reads for gap alignment by BWA (-e 30) to detect indels (small Insertion/Deletion). Then we used SAMtools pileup to detect the indels from mapping reads with gap. Gaps that were supported by at least 3 paired-end reads were extracted. Inversions and other additional structural variants were identified with BreakDancer vsn 1.1 (Chen et al., 2009) and filtered based on the BWA quality score. We did not find any fixed inversion among species in the *H. cydno – H. pachinus* divergent regions (Table S4).

GO term enrichment analysis

To test for enrichment of specific gene ontology categories in divergent regions of the genome, gene sequences falling within C/P, C/M, and P/M regions were extracted from the Hmel1.1 gene annotations. Because the current gene ontology terms associated with Hmel1.1 are sparsely assigned, we blasted Hmel1.1 gene against known genes in the *Drosophila melanogaster* (Dmel Release 5.48, FlyBase). Hmel genes that blasted to Dmel genes with e-values < 0.05 were considered matched. We then used appropriate FlyBase accessions (e.g., accessions within vs. accessions outside of divergent regions) as the input for GO Elite (http://www.genmapp.org/go_elite/). We combined permuted probabilities from the merged GO Elite analysis for the three comparisons using Fisher's method and then adjusted the tests for multiple comparisons[12] based on the total number of genes in the comparison set, multiplied by 3 to further correct for the three non-independent comparisons (C/P, C/M & P/M) this method identifies GO terms enriched across all three comparisons (Table S6).

Supplemental References

Chen, K., Wallis, J.W., McLellan, M.D., Larson, D.E., Kalicki, J.M., Pohl, C.S., McGrath, S.D., Wendl, M.C., Zhang, Q.Y., Locke, D.P.*, et al.* (2009). BreakDancer: an algorithm for high-resolution mapping of genomic structural variation. Nat. Methods *6*, 677-681.
